# Multi-omics Analyses Reveal Synergistic Carbohydrate Metabolism in Streptococcus mutans-Candida albicans Mixed-Species Biofilms

**DOI:** 10.1128/IAI.00339-19

**Published:** 2019-09-19

**Authors:** K. Ellepola, T. Truong, Y. Liu, Q. Lin, T. K. Lim, Y. M. Lee, T. Cao, H. Koo, C. J. Seneviratne

**Affiliations:** a Oral Sciences, Faculty of Dentistry, National University of Singapore, Singapore; b Biofilm Research Labs, Levy Center for Oral Health, Department of Orthodontics and Divisions of Pediatric Dentistry & Community Oral Health, School of Dental Medicine, University of Pennsylvania, Philadelphia, Pennsylvania, USA; c Protein and Proteomic Centre, Department of Biological Sciences, National University of Singapore, Singapore; d Center of Oral and Craniofacial Biology, School of Dentistry, Louisiana State University Health Sciences Center, New Orleans, Louisiana, USA; e National Dental Centre Singapore, Oral Health ACP, SingHealth Duke NUS, Singapore; University of Cincinnati

**Keywords:** *Candida albicans*, early-childhood caries, omics, *Streptococcus mutans*, mixed-species biofilms

## Abstract

Candida albicans, a major opportunistic fungal pathogen, is frequently found together with Streptococcus mutans in dental biofilms associated with severe childhood caries (tooth decay), a prevalent pediatric oral disease. However, the impact of this cross-kingdom relationship on C. albicans remains largely uncharacterized. Here, we employed a novel quantitative proteomics approach in conjunction with transcriptomic profiling to unravel molecular pathways of C. albicans when cocultured with S. mutans in mixed biofilms. RNA sequencing and iTRAQ (isobaric tags for relative and absolute quantitation)-based quantitative proteomics revealed that C. albicans genes and proteins associated with carbohydrate metabolism were significantly enhanced, including sugar transport, aerobic respiration, pyruvate breakdown, and the glyoxylate cycle. Other C. albicans genes and proteins directly and indirectly related to cell morphogenesis and cell wall components such as mannan and glucan were also upregulated, indicating enhanced fungal activity in mixed-species biofilm. Further analyses revealed that S. mutans-derived exoenzyme glucosyltransferase B (GtfB), which binds to the fungal cell surface to promote coadhesion, can break down sucrose into glucose and fructose that can be readily metabolized by C. albicans, enhancing growth and acid production. Altogether, we identified key pathways used by C. albicans in the mixed biofilm, indicating an active fungal role in the sugar metabolism and environmental acidification (key virulence traits associated with caries onset) when interacting with S. mutans, and a new cross-feeding mechanism mediated by GtfB that enhances C. albicans carbohydrate utilization. In addition, we demonstrate that comprehensive transcriptomics and quantitative proteomics can be powerful tools to study microbial contributions which remain underexplored in cross-kingdom biofilms.

## Abstract (precis)

Candida albicans, a major opportunistic fungal pathogen, is frequently found together with Streptococcus mutans in dental biofilms associated with severe childhood caries (tooth decay), a prevalent pediatric oral disease. However, the impact of this cross-kingdom relationship on C. albicans remains largely uncharacterized. Here, we employed a novel quantitative proteomics approach in conjunction with transcriptomic profiling to unravel molecular pathways of C. albicans when cocultured with S. mutans in mixed biofilms.

## INTRODUCTION

Early-childhood caries (ECC) is an aggressive form of dental caries (tooth decay) afflicting toddlers of lower socioeconomic status (1), and it remains one of the most prevalent and costly pediatric diseases worldwide (2, 3). ECC is characterized by a heavy microbial carriage forming virulent and intractable plaque biofilms on teeth exposed to sugar-rich dietary habits (2, 4, 5), which cause rampant tooth destruction with significant consequences for the general health and well-being of the affected children (4). Thus, enhanced understanding of the microbiological factors associated with this highly prevalent pediatric oral disease is needed.

Streptococcus mutans, a biofilm-forming acidogenic bacterium in the oral cavity, is considered an important bacterial pathogen associated with ECC. Enhanced accumulation of S. mutans in ECC plaque biofilms has been attributed to protracted feeding of dietary sugars such as sucrose (6–8). S. mutans is uniquely capable of utilizing sucrose to produce both acids and insoluble exopolysaccharides (EPS) to help develop cariogenic biofilms characterized by an acidic milieu and adhesive structure, causing damage to the mineralized tooth tissue (5, 9). In particular, S. mutans secretes exoenzymes called glucosyltransferases (Gtfs) that convert sucrose into extracellular glucans, a major constituent of the EPS. This extracellular glucan enhances the bacterial adhesion to the tooth surface and aids in bacterial coaggregation with other species, leading to the development of cariogenic biofilms (10).

Although ECC has been associated with cariogenic bacteria (5, 11), Candida albicans (a major oral fungal organism) is often detected in high numbers with elevated levels of S. mutans in biofilms from children with severe ECC (12–16). The presence of sucrose is a key environmental factor that significantly enhances the coadhesion and coexistence between these organisms (17–19). On further examination, it has been found that C. albicans interacts with S. mutans by enhancing EPS production and microbial carriage of both organisms within mixed biofilms (17, 20). Initial mechanistic studies revealed that S. mutans-derived GtfB binds firmly to the cell wall surface of C. albicans and produces large amounts of EPS α-glucans in the presence of sucrose (21, 22). These glucans serve as binding sites for S. mutans, promoting coadhesion and bacterial-fungal accumulation (21, 23). Interestingly, we also observed that GtfB was able to enhance the biofilm formation of a *bcr1Δ*/Δ mutant, which is canonically defective in biofilm development (20). Importantly, this cross-kingdom interaction resulted in a highly virulent mixed biofilm leading to rampant caries in a rodent model of ECC (17). The presence of C. albicans enhanced the accumulation of viable S. mutans cells and influenced the bacterial transcriptome, inducing the expression of key metabolic and virulence genes (17). These findings are consistent with clinical findings suggesting an active role for C. albicans in the context of severe ECC (18). However, how C. albicans responds in this cariogenic biofilm environment with S. mutans and the impact of transcriptomic changes at the protein level remain unclear.

Here we employed recently developed iTRAQ (isobaric tags for relative and absolute quantitation)-based quantitative proteomics complemented by RNA sequencing (RNA-Seq)-based transcriptomics to assess the molecular pathways mediating C. albicans and its interactions with S. mutans in this virulent cross-kingdom relationship. We present a comprehensive transcriptome-proteome approach to study the contribution of each of the organisms within mixed biofilms, which remains mostly uncharacterized in cross-kingdom biofilms. The multi-omics approach coupled with gene ontology (GO) pathway analysis and biochemical methods reveals that C. albicans has an active role in the sugar metabolism and environmental acidification when interacting with S. mutans. Conversely, the presence of C. albicans also modulated the S. mutans proteome related to carbohydrate utilization and glucan biosynthesis. Furthermore, we found an intriguing cooperative mechanism by which the bacterial GtfB can directly contribute to C. albicans growth and metabolism by providing glucose and fructose from sucrose breakdown. Altogether, the present study provides new insights into the synergistic cross-kingdom interaction between S. mutans and C. albicans within biofilms and a new cross-feeding role for GtfB in the context of ECC. These findings indicate the importance of developing therapeutic strategies targeting fungal contributions and bacterial interactions associated with a prevalent childhood disease.

## RESULTS

### Candida albicans transcriptomic changes in mixed biofilm with Streptococcus mutans.

There were 6,081 C. albicans genes that were expressed in both single-species and mixed-species biofilms, 169 genes expressed only in C. albicans mixed-species biofilm, and 19 genes expressed only in C. albicans single-species biofilms. Conversely, 1,968 genes in S. mutans were expressed in both single- and mixed-species biofilms, while 9 genes were expressed only in S. mutans single-species biofilms.

**FIG 1:**
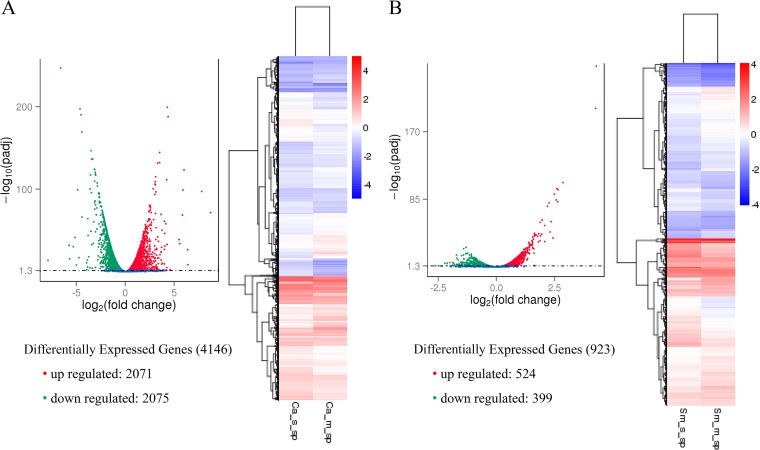
Differential expression of microbial genes. Volcano plots and heat maps illustrating the differential expression of C. albicans (A) and S. mutans (B) genes in mixed-species biofilms compared with the single-species biofilm counterparts.

It was observed that out of 6,384 identified genes in C. albicans, 4,146 genes were differentially expressed (Fig. 1A; see Tables S1 and S2 in the supplemental material). Among the differentially expressed genes, 2,071 (32.44%) genes were significantly upregulated and 2,075 genes (32.50%) were downregulated based on the *P* value cutoff applied, with statistical significance at a *P* value of *≤*0.05. Another filtering technique was used to identify the most highly upregulated or downregulated genes based on the log_2_ fold change. All the genes with a log_2_ fold change of more than 1 were considered to be upregulated, and all genes with a log_2_ fold change of less than −1 were considered to be downregulated. Using this technique, 958 upregulated genes and 873 downregulated genes in C. albicans were identified.

Importantly, Candida albicans genes associated with carbohydrate metabolism were remarkably enhanced in mixed biofilms. Several genes associated with sugar transportation systems in C. albicans showed enhanced expression in GO pathway analysis (see Fig. 3A). *SNF3* (W5Q_03002) expression was enhanced; this gene encodes Snf3p in Saccharomyces cerevisiae, which is analogous to the Hgt4 protein of C. albicans (orf19.5962), and these proteins act as glucose sensors and govern sugar acquisition by regulating the expression of genes encoding hexose transporters. *HGT4* expression is repressed by high levels of glucose, suggesting that it may act as a high-affinity sugar sensor. Glucose sensing through Hgt4 also affects the yeast-to-hyphal morphological switch of C. albicans cells (24). Interestingly, *ITR1* (W5Q_02514), which is important for the acquisition of inositol (25), and *ITR2* (W5Q_01924), which is associated with potential sugar transporter activity, were also induced. In addition, various pathways associated with sugar metabolism of C. albicans were affected. For instance, *GAL1* (W5Q_00201), which encodes a galactokinase similar to S. cerevisiae GAL3 associated with galactose metabolism, *ARA1* (W5Q_02234D), encoding arabinose dehydrogenase involved in arabinose metabolism, and *TPS3* (W5Q_02484), encoding a regulatory subunit of trehalose-6-phosphate synthase involved in trehalose metabolism, were upregulated.

**FIG 2:**
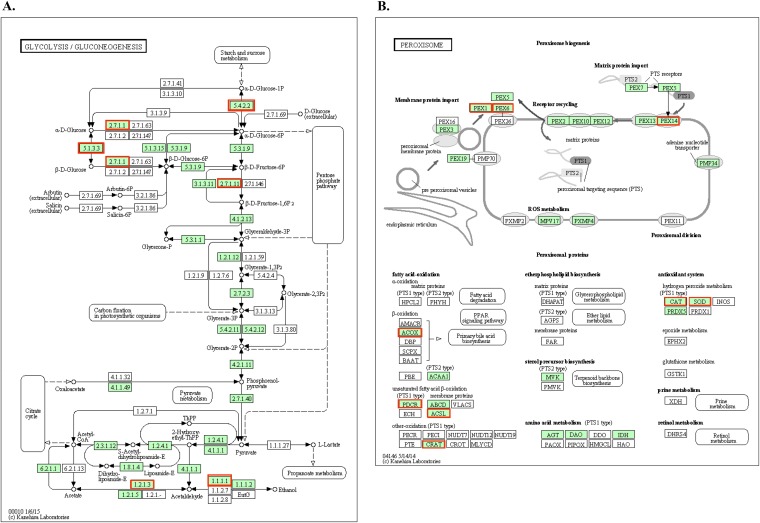
KEGG pathway analysis of C. albicans upregulated genes. C. albicans glycolysis/gluconeogenesis pathways (A) and peroxisomal assembly and fatty acid β-oxidation pathways (B) were upregulated in S. mutans-C. albicans mixed-species biofilms. The green boxes represent the C. albicans-specific gene entries identified in the reference pathway, and the red boxes represent the genes that were significantly upregulated in these specific pathways.

Moreover, genes associated with glycolysis and pyruvate degradation to ethanol and acetate production were upregulated in C. albicans in the mixed-species biofilms (Fig. 2A). These include *GPM1* (W5Q_00421), encoding the protein phosphoglycerate mutase, which is involved in the third step of the subpathway that synthesizes pyruvate from d-glyceraldehyde 3-phosphate in glycolysis. Other genes involved in the tricarboxylic acid cycle, such as *FUMH* (W5Q_03272), which encodes a fumarate hydratase, were also induced. Genes associated with the electron transport chain, such as *CYC2* (W5Q_04301), encoding a cytochrome *c* mitochondrial import factor, as well as *COX15* (W5Q_04661), which encodes cytochrome *c* oxidase assembly protein (COX15) involved in biosynthesis of heme A during cellular respiration, were upregulated. In addition, genes *PDP1* (W5Q_06274) and *PDC2* (W5Q_00962), associated with pyruvate metabolism, were upregulated. *PDP1* encodes a pyruvate dehydrogenase phosphatase, whereas *PDC2* (W5Q_00962) is essential for the synthesis of pyruvate decarboxylase and encodes a transcriptional regulator for pyruvate decarboxylase.

Important genes associated with lactic acid metabolism were also upregulated in the C. albicans transcriptome. For instance, *CYB2* (W5Q_01316), encoding Cyb2, a heme-containing dehydrogenase (l-lactate cytochrome *c* oxidoreductase) essential for the utilization of l-lactate as a carbon source, and *DLD1* (W5Q_01734), which encodes a putative d-lactate dehydrogenase, were among the upregulated genes. Several genes required for peroxisomal assembly and fatty acid β-oxidation were found to be upregulated in C. albicans in mixed-species biofilms (Fig. 2B; Table S1). Genes associated with growth on ethanol and acetate were also induced. These include *YAT1* (W5Q_00164), which contributes to the transport of acetyl coenzyme A (acetyl-CoA) from the cytosol during growth on ethanol or acetate. *LPG20* (W5Q_00403), encoding a putative aryl alcohol dehydrogenase, *SAD3* (W5Q_01879), encoding one of a tandem pair of alcohol dehydrogenases, and *ADH2* (W5Q_00801), encoding an alcohol dehydrogenase 2 which catalyzes the conversion of ethanol to acetaldehyde, were also upregulated. Altogether, the presence of S. mutans appears to significantly increase C. albicans carbohydrate transport and metabolic processes in mixed-species biofilms.

**FIG 3:**
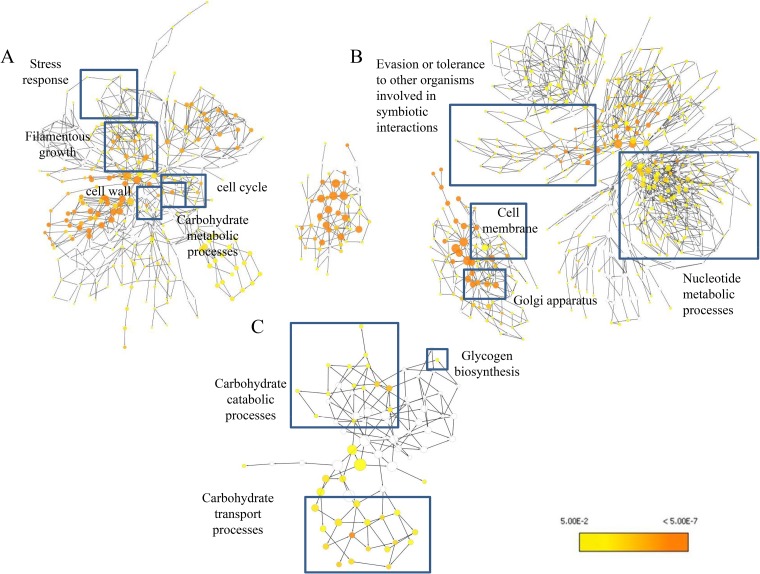
Gene ontology pathway analysis. GO pathway maps for C. albicans upregulated genes (A), C. albicans downregulated genes (B), and S. mutans upregulated genes (C) are shown. Uncolored nodes represent GO categories that are not overrepresented. Yellow nodes represent GO categories that are overrepresented at a significant level. The node color becomes increasingly more orange when the *P* values are more significant.

The presence of S. mutans also enhanced C. albicans genes involved in hyphal formation and cell wall properties in the mixed-species biofilm (Tables S1 and S2). Genes involved in transcriptional regulation related to the filamentous growth of the fungi and genes associated with cell wall components such as mannans and glucans were upregulated. The GO ontology pathway analysis showed the involvement of the aforementioned upregulated genes associated with key factors in *Candida* biology and virulence (Fig. 3A). Furthermore, genes associated with ergosterol biosynthesis, vacuolar development, biofilm dispersal, cation uptake and transport, heat shock proteins, drug transport, and cellular stress and genes associated with mitotic and meiotic cell division were also upregulated (Table S1).

Conversely, several C. albicans genes were downregulated within mixed biofilms. Genes associated with outer cell membranes, vacuolar membranes, endoplasmic reticulum, and Golgi apparatus membranes were repressed (Fig. 3B; Tables S1 and S2). These genes encode integral structural proteins, translocases, or permeases, indicating a possible role in regulating membrane transport and homeostasis. However, many downregulated proteins either were unknown or had an unclear functional role associated with the mixed-species biofilm condition, requiring further investigation.

C. albicans also significantly altered the transcriptomic profile of S. mutans in mixed-species biofilms, consistent with previous findings (26). RNA sequencing demonstrated that S. mutans genes associated with the phosphotransferase system (PTS), ABC sugar transporter system, and carbohydrate metabolism, as well as glycogen biosynthesis, were significantly upregulated (*P < *0.05) (see Table S3 in the supplemental material). GO pathway analysis confirmed these observations (Fig. 3C).

### iTRAQ-based quantitative proteomics of Candida albicans and Streptococcus mutans in mixed-species biofilms.

To determine the impact of transcriptomic changes at the protein level, we performed an iTRAQ-based mass spectrometry analysis to study the proteomic changes of each species in the S. mutans-C. albicans mixed-species biofilms compared to the single-species biofilms. A schematic representation of the experimental design for iTRAQ analysis is shown in Fig. S1A and B in the supplemental material. The methodologies used to identify the proteins with altered abundance in S. mutans and C. albicans in mixed-species biofilm samples compared to the single-species biofilms are illustrated in Fig. S2 and S3 in the supplemental material, respectively.

**FIG 4:**
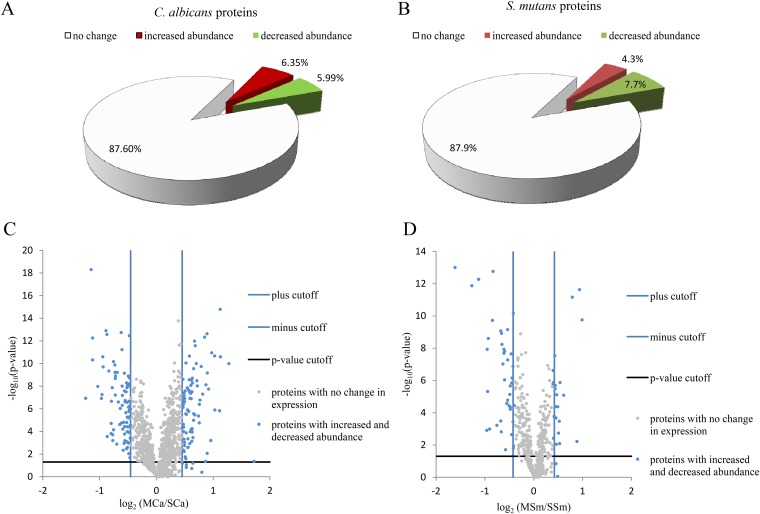
Expression of C. albicans and S. mutans proteins in the mixed-species biofilm. (A and B) Pie charts showing percentage of the number of proteins showing “no change,” “increased abundance,” or “decreased abundance” in the mixed-species biofilm versus single-species biofilm of C. albicans (A) and S. mutans (B). (C and D) Volcano plots showing the distribution of the expression of C. albicans (C) and S. mutans (D) proteins in the mixed-species biofilm compared to single-species biofilms. The fold cutoff values (blue lines) are indicated, along with the *P* value cutoff (black lines).

Proteins that qualified to all the cutoff values in the respective data sets with a significant *P* value were chosen as significantly altered proteins. After comprehensive and stringent calculations, a total of 175 C. albicans proteins (see Table S4 in the supplemental material) and 66 S. mutans proteins (see Table S5 in the supplemental material) were identified as altered proteins. Out of 175 C. albicans proteins, 91 proteins increased in abundance and 84 proteins decreased in abundance. Of 66 S. mutans proteins, 25 proteins increased in abundance and 41 proteins decreased in abundance. Finally, after considering the *P* values, out of 1,384 C. albicans proteins, 88 proteins (6.35%) increased in abundance and 83 proteins (5.99%) decreased in abundance (Fig. 4A). For S. mutans, out of 530 proteins, 23 proteins (4.3%) increased in abundance and 41 proteins (7.7%) decreased in abundance (Fig. 4B). The expression patterns of these proteins are illustrated using volcano plots (Fig. 4C and D). The complete lists of C. albicans and S. mutans proteins with altered abundance are shown in Tables S4 and S5 in the supplemental material, respectively.

C. albicans proteins associated with carbohydrate metabolism, such as alpha-1,4-glucan phosphorylase, hexokinase-2, isocitrate lyase, and malate synthase, significantly increased in abundance in mixed-species biofilms. Among these proteins, alpha-1,4-glucan phosphorylase is an important allosteric enzyme for carbohydrate metabolism (27). Hexokinase-2, the main glucose-phosphorylating enzyme in C. albicans, increased in abundance, indicating active glucose utilization (28). Isocitrate lyase and malate synthase are two important enzymes in the glyoxylate cycle and fatty acid β-oxidation pathways converting fatty acids into glucose. Proteins associated with lactic acid production (glyoxalase, putative NADPH-dependent methylglyoxal reductase, and Cyb2) and formic acid oxidation (formate dehydrogenase) increased in abundance, suggesting acid accumulation via carbohydrate breakdown.

**FIG 5:**
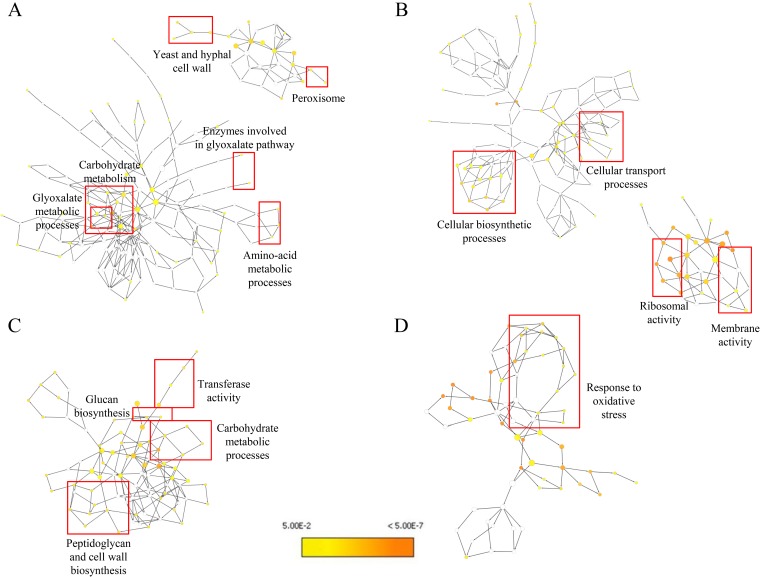
Gene ontology pathway analysis for the proteins with changed abundance. (A and B) Gene ontology pathways constructed using the Cytoscape software and the Bingo plugin show pathways affected in C. albicans based on proteins with increased (A) and decreased (B) abundance. (C and D) Similarly, pathways affected in S. mutans are shown for proteins with increased (C) and decreased (D) abundance. Uncolored nodes represent GO categories that are not overrepresented. Yellow nodes represent GO categories that are overrepresented at a significant level. The node color becomes increasingly more orange when the *P* values are more significant.

Proteins associated with invasive growth, filament formation, dispersal, and pathogenesis, such as pH-responsive protein-2, yeast-form wall protein 1 (YWP1), Fma1p, Hsp21, Hsp12, and Hsp31, were also detected in higher abundance (versus that in single-species biofilm). In addition, Cytoscape was used to perform pathway analysis for the proteins with altered abundance (Fig. 5). Notable upregulated pathways in the C. albicans proteome in the mixed-species proteome compared to the single-species proteome include carbohydrate metabolism, glyoxylate pathway, and cell wall proteins (Fig. 5A), while cellular biosynthetic and transport processes were downregulated (Fig. 5B).

On the other hand, S. mutans proteins in the mixed-species biofilm proteome indicated that carbohydrate metabolism, glucan biosynthesis and transferase activity, and peptidoglycan and cell wall biosynthesis were significantly increased (versus those in single-species biofilm) (Fig. 5C). S. mutans carbohydrate metabolism-associated proteins, particularly proteins in the tricarboxylic acid cycle such as citrate synthase (CitZ) and proteins in the pentose phosphate pathway such as ribose-5-phosphate isomerase A (RpiA), increased in abundance. Formate acetyltransferase, which is involved in pyruvate fermentation, was significantly more abundant in the S. mutans mixed-biofilm proteome. Proteins in the PTS sugar transport system (PTS system) (mannose-specific IID component protein and HPr kinase/phosphorylase protein) and ABC sugar transport system (putative ABC transporter, ATP-binding protein amino acid transport system proteins) significantly increased in abundance. S. mutans sucrose phosphorylase/GtfA enzyme showed increased abundance in the mixed-species biofilm, demonstrating the enhanced activity of glucan biosynthesis and transferase activity (Fig. 5C). Oxidative stress response pathways were suppressed in S. mutans in the mixed-species biofilms compared to the S. mutans single-species biofilms (Fig. 5D).

### Similarities and differences in the RNA-sequencing and iTRAQ proteomics data.

Independently analyzing the gene and protein level expression and GO ontology pathway analysis showed a high level of similarity between the transcriptomics and proteomics data (Tables S2 to S5; Fig. 3 and 5). Particularly, C. albicans genes and proteins associated with carbohydrate metabolism, sugar transport systems, glycolysis, pyruvate degradation to ethanol and acetate production, the tricarboxylic acid cycle, and the electron transport chain were significantly affected at both the transcription and protein levels. Glyoxylate cycle and fatty acid β-oxidation pathways were similarly affected. The expression of genes and proteins associated with cell wall and hyphal growth in C. albicans was enhanced in mixed biofilms. Both transcriptomic and proteomic data suggest the enhanced ability of C. albicans to produce acids such as lactic acid and formic acid under mixed-species biofilm conditions. Conversely, there were dissimilarities between the transcriptomics and proteomics data related to stress response, amino acid metabolic processes, nucleotide metabolic processes, and cell cycle-related processes in C. albicans. For S. mutans, both transcriptomics and proteomics data showed a remarkable similarity in enhanced expression of carbohydrate metabolic processes, carbohydrate transport processes, and glucan biosynthesis. However, there were differences related to transferase activity and stress response.

### Cell-free supernatants of single and mixed biofilms enhance Candida albicans biofilm formation.

**FIG 6:**
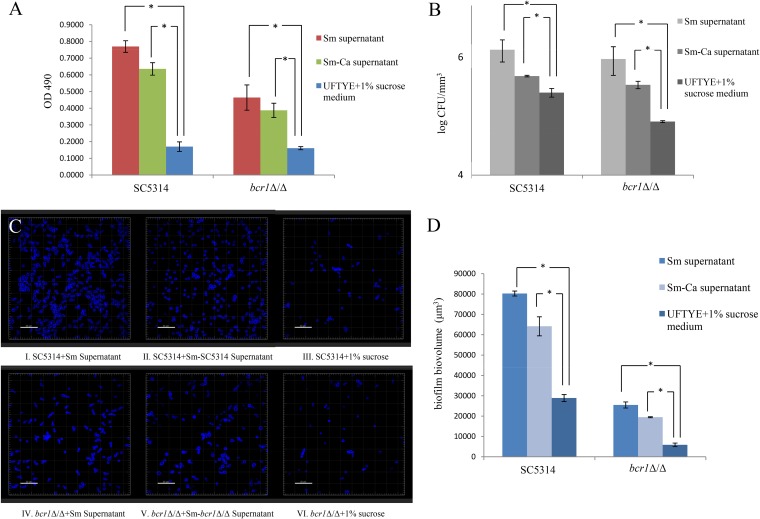
Comparison of C. albicans biofilm formation in cell-free supernatants. C. albicans biofilms were formed in the presence of S. mutans (Sm) and S. mutans-C. albicans (Sm-Ca) cell-free biofilm supernatants. (A to C) Biofilm formation was evaluated using XTT reduction assay (A), CFU counting (B), and CLSM (C). (D) Biofilm biovolumes were evaluated using Imaris software. The C. albicans SC5314 and *bcr1*Δ/Δ strains showed more biofilm in S. mutans supernatant and S. mutans-C. albicans supernatant than in the UFTYE–1% sucrose medium. Biofilm formation in the S. mutans supernatant was slightly higher than that in the S. mutans-C. albicans supernatant.

Transcriptomics and proteomics data indicated a significant increase in C. albicans and S. mutans carbohydrate metabolism in mixed-species biofilms. Thus, the composition of carbohydrates in the mixed-species S. mutans-C. albicans biofilm supernatant may be altered compared to that in the single-species biofilm supernatant. We used S. mutans and S. mutans-C. albicans cell-free biofilm supernatants to evaluate the effect on C. albicans biofilm formation. A 2,3-bis-(2-methoxy-4-nitro-5-sulfophenyl)-2H-tetrazolium-5-carboxanilide salt (XTT) assay demonstrated that biofilm formation by C. albicans was significantly lower in 1% sucrose medium than that in the supernatants of S. mutans and S. mutans-C. albicans biofilms (Fig. 6A). A C. albicans *bcr1*Δ/Δ mutant also showed significantly lower biofilm formation when grown in 1% sucrose medium than the S. mutans *bcr1*Δ/Δ supernatant and the S. mutans single-species biofilm supernatant. CFU counting (Fig. 6B) further confirmed the results, showing a similar pattern of increase in the number of viable cells when treated with different supernatants. Confocal laser scanning microscopy (CLSM) imaging (Fig. 6C) and subsequent evaluation of biovolumes using Imaris software (Fig. 6D) corroborated the observations from XTT assay and CFU counting. Taking the results together, it appeared that C. albicans formed more biofilms when treated with S. mutans single-species biofilm supernatant or S. mutans-C. albicans mixed-species biofilm supernatant than in the ultrafiltered tryptone-yeast extract (UFTYE)–1% sucrose medium.

### Glucosyltransferase B increases Candida albicans growth in mixed-species biofilms.

**FIG 7:**
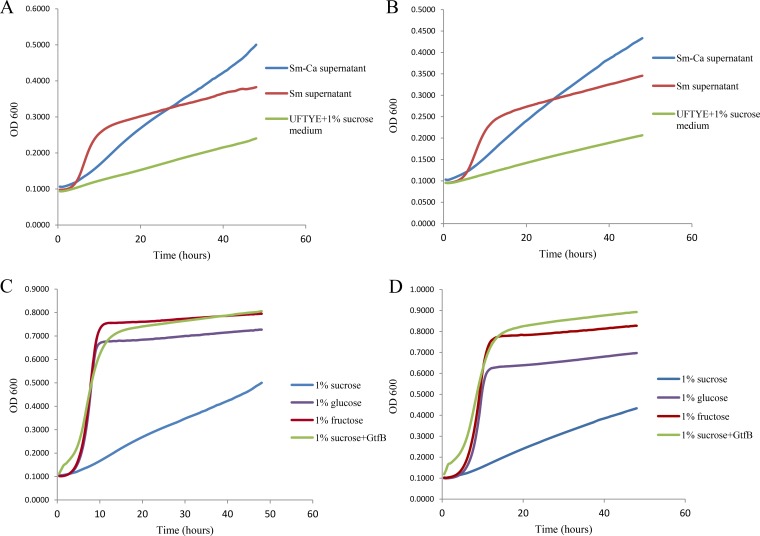
C. albicans growth kinetics. (A and B) Growth kinetic assays of the C. albicans SC5314 (A) and *bcr1*Δ/Δ (B) strains showed rapid growth when S. mutans supernatant was used. C. albicans in UFTYE–1% sucrose medium showed the lowest growth rate, while *Candida* in S. mutans-C. albicans supernatants showed a moderate growth rate (relative to that in S. mutans supernatant). (C and D) Growth kinetics of the SC5314 (C) and *bcr1*Δ/Δ (D) strains were evaluated in UFTYE medium containing 1% sucrose, 1% sucrose plus GtfB, 1% glucose, or 1% fructose. Both strains showed slow growth in UFTYE–1% sucrose medium. Addition of GtfB in UFTYE–1% sucrose significantly increased the growth (versus that with 1% sucrose). Rapid growth of the two strains was observed when they were incubated in UFTYE containing 1% glucose or 1% fructose.

We hypothesized that the impact on C. albicans biofilm formation was due to enhanced sugar availability in the S. mutans and S. mutans-C. albicans supernatants. To test this hypothesis, we evaluated the growth kinetics of C. albicans in medium containing either S. mutans biofilm supernatant or S. mutans-C. albicans biofilm supernatant. Interestingly, the SC5314 (Fig. 7A) and *bcr1*Δ/Δ (Fig. 7B) strains showed a rapid increase in C. albicans growth when S. mutans supernatant was used. In contrast, C. albicans showed a lower growth rate in S. mutans-C. albicans supernatant. Moreover, growth of C. albicans in UFTYE–1% sucrose medium was slower than that with both S. mutans and S. mutans-C. albicans supernatants. This implies that a factor in the S. mutans supernatant supported C. albicans growth by converting sucrose (which is not efficiently metabolized by *Candida*) into monosaccharides, which can be rapidly utilized by the fungal cells.

We assumed that GtfB secreted by S. mutans to the medium may be one of the components responsible for the above observation given its key role in this bacterial-fungal interaction and its catalytic activity on sucrose (17, 22). Therefore, the growth kinetics of C. albicans SC5314 (Fig. 7C) and the *bcr1*Δ/Δ strain (Fig. 7D) were evaluated in UFTYE medium containing 1% sucrose, 1% sucrose supplemented with GtfB, 1% glucose, or 1% fructose. A rapid increase in the growth of C. albicans was observed when it was grown in UFTYE containing 1% glucose or 1% fructose. Comparatively, the growth of C. albicans in UFTYE–1% sucrose medium was slower. However, introducing GtfB into the UFTYE–1% sucrose medium significantly increased the growth rates of the SC5314 and *bcr1*Δ/Δ strains to a level similar to that found in UFTYE medium containing 1% glucose or 1% fructose.

### Glucosyltransferase B-mediated breakdown of sucrose into glucose and fructose enhances Candida albicans growth.

**FIG 8:**
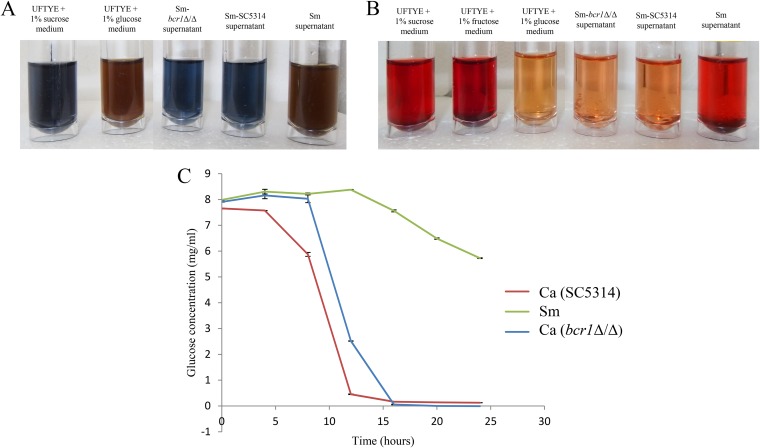
Detection of sugar in supernatants and rates of glucose consumption by S. mutans and C. albicans. (A) Benedict’s assay showed that S. mutans supernatant contains reducing sugars (e.g., glucose and fructose), whereas these carbohydrates were not detected in either S. mutans *bcr1*Δ/Δ supernatant or S. mutans SC5314 supernatant. (B) Seliwanoff’s test showed the presence of keto-sugars (e.g., fructose) in the S. mutans supernatant. The S. mutans *bcr1*Δ/Δ supernatant and S. mutans SC5314 supernatant showed no presence of keto-sugars. (C) C. albicans SC5314 and *bcr1*Δ/Δ displayed a significantly higher glucose consumption rate than S. mutans (C).

We reasoned that S. mutans-derived GtfB, in addition to glucan synthesis, can also release significant amounts of glucose and fructose from sucrose breakdown, allowing C. albicans to utilize the monosaccharides. Hence, we performed Benedict’s test for the detection of reducing sugars such as glucose and fructose in the S. mutans and S. mutans-C. albicans supernatants (Fig. 8A). The S. mutans supernatant tested positive for reducing sugars. In contrast, S. mutans-C. albicans SC5314 and S. mutans-C. albicans *bcr1Δ*/Δ supernatants were devoid of significant amounts of reducing sugars. Seliwanoff’s test was used to detect the keto-sugars, such as fructose, in the cell-free supernatants (Fig. 8B). The test showed positive results for keto-sugars in S. mutans supernatant, indicating the presence of fructose, but we were unable to detect fructose in the supernatant of mixed-species biofilms of either S. mutans-C. albicans SC5314 or S. mutans-C. albicans *bcr1*Δ/Δ. Furthermore, using a commercial glucose assay kit, we found that C. albicans utilizes glucose at a higher rate than S. mutans (Fig. 8C). Given the reduced amounts of glucose and fructose in the mixed-biofilm supernatant, the data suggest enhanced utilization of the aforementioned monosaccharides when C. albicans is grown in the presence of S. mutans. This is an important finding to describe the nature of this bacterial-fungal interaction in mixed-species biofilms.

### The presence of Candida albicans contributes to lowering the pH in mixed-species biofilm.

**FIG 9:**
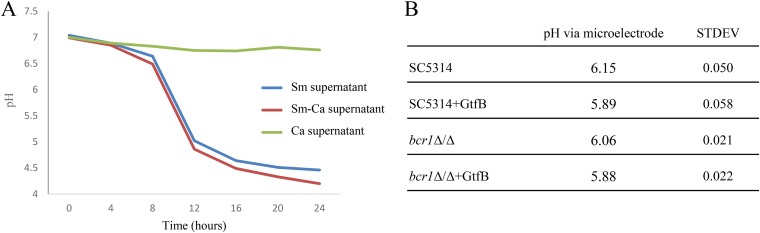
pH measurements in S. mutans-C. albicans mixed-species biofilms. (A) The pH in biofilm supernatants was measured in a time-dependent manner. In comparison to the UFTYE–1% sucrose medium, the S. mutans supernatant had significantly lower pH values. The S. mutans-C. albicans supernatant displayed the lowest pH values. (B) The pH of biofilm microenvironments of the SC5314 and *bcr1*Δ/Δ strains in the presence of GtfB was measured using a microelectrode (*n* = 4). The presence of GtfB lowered the pH of the SC5314 and *bcr1*Δ/Δ biofilm microenvironments.

To further examine the implication of above observation on the microenvironment of mixed-species biofilms, the pHs of the supernatants of single and mixed-species biofilms were measured in a time-dependent manner (Fig. 9A). The supernatants of S. mutans single-species biofilms and S. mutans-C. albicans mixed-species biofilms had a significant reduction in pH after 24 h compared to that of the UFTYE–1% sucrose medium. The pH of the supernatant of S. mutans-C. albicans mixed-species biofilm was slightly lower than that of the S. mutans single-species biofilm supernatant, suggesting that C. albicans in the mixed-species biofilm can contribute, to some extent, to lowering the pH. Considering that S. mutans-derived GtfB converts sucrose into monosaccharides, it is possible that C. albicans can utilize these sugars to further reduce the pH. We measured the *in situ* pH of C. albicans biofilms using a microelectrode upon supplementation with GtfB. The addition of GtfB lowered the pH of the SC5314 and *bcr1*Δ/Δ biofilm microenvironments by approximately 0.3 and 0.2 pH units compared to that of the controls without GtfB supplementation (Fig. 9B). Hence, it appears that C. albicans can also contribute with the acidification of the environmental pH in mixed biofilms with S. mutans, at least in part via a GtfB-mediated mechanism.

## DISCUSSION

A unique microbiological feature of severe ECC is the frequent isolation of C. albicans together with heavy carriage of S. mutans in biofilms on the teeth of diseased pediatric patients (12, 14–16, 29). Previous studies demonstrated that these organisms can synergistically interact and enhance the accumulation of each species within mixed biofilms (17, 20, 29). A quantitative proteomics approach coupled with RNA-Seq and systems analysis was employed to generate detailed molecular pathways associated with this cross-kingdom biofilm interaction, with a particular focus on the C. albicans counterpart, which remains underexplored in a cariogenic setting. We identified key pathways used by C. albicans in the mixed biofilm that indicate an active fungal role in the sugar metabolism and environmental acidification as well as biofilm architecture when interacting with S. mutans under cariogenic conditions. Conversely, S. mutans PTS and ABC sugar transporter systems and carbohydrate metabolism were also enhanced in mixed biofilm with C. albicans, consistent with previous findings (26). In addition, we found a new cross-feeding mechanism mediated by GtfB that benefits C. albicans by providing readily metabolizable monosaccharides, promoting fungal growth, and lowering the environmental pH, which in turn can favor S. mutans survival. The data show that C. albicans develops a symbiotic coexistence with S. mutans in a sucrose-rich environment, enhancing the bacterial-fungal sugar metabolism and acid production that are associated with caries onset.

Combined RNA-Seq based-transcriptomics and iTRAQ quantitative proteomics demonstrated enhanced expression of C. albicans genes and proteins related to carbohydrate metabolism when cocultured with S. mutans, including sugar transport systems, glycolysis, pyruvate degradation to ethanol and acetate production, the tricarboxylic acid cycle, and the electron transport chain. Glyoxylate cycle and fatty acid β-oxidation pathways were also enhanced, which act as anaplerotic routes for replenishing pyruvate for the tricarboxylic acid cycle. Paradoxically, C. albicans poorly utilizes sucrose (30), indicating that the presence of S. mutans can facilitate sugar transport and metabolism. C. albicans showed a higher initial growth rate when cultured in S. mutans biofilm supernatant. Further carbohydrate analysis indicated the presence of glucose and fructose in the supernatant, which implies that S. mutans has the ability to cross-feed the sucrose breakdown products to C. albicans, as suggested in other studies (26, 31, 32). Indeed, C. albicans grows at much higher rate when cultured in glucose or fructose than in sucrose (Fig. 7C and D), supporting the importance of cross-feeding for fungal survival under cariogenic conditions.

Next, we postulated that the S. mutans GtfB exoenzyme could be a key contributor for converting sucrose to glucose and fructose. Previous studies have shown that S. mutans-secreted GtfB binds with higher affinity and avidity to C. albicans cell wall than to its own bacterial surface (21, 31). Hence, the breakdown of sucrose into glucose and fructose may occur in close proximity to the fungal surface microenvironment, allowing C. albicans to utilize the monosaccharides efficiently, which could explain the increased C. albicans growth in mixed-species biofilms (20). Our data revealed that S. mutans GtfB is capable of breaking down sucrose in the culture medium into monosaccharides. Excitingly, supplementing GtfB enzyme into the sucrose medium significantly boosted the growth of C. albicans as a single species, reaching a rate similar to that found when the fungal cells were grown in glucose and fructose (Fig. 7C and D). These findings indicate that S. mutans cross-feeding, mediated at least in part by GtfB, can compensate for the inefficiency of C. albicans to utilize sucrose, boosting its ability to accumulate under cariogenic conditions.

Increased sugar utilization by C. albicans with enhanced S. mutans sugar transport and carbohydrate metabolism in mixed biofilm resulted in a biologically important consequence. Mixed-species biofilms were able to further reduce the culture pH compared to that of S. mutans single-species biofilms. C. albicans appears to contribute to lowering the pH of the mixed-species biofilm. Transcriptomic and proteomic data support the ability of C. albicans to produce acids such as lactic acid and formic acid, consistent with previous findings showing enhanced sugar utilization and levels of extracellular lactate and formate in S. mutans-C. albicans mixed-species biofilm supernatant via H^1^ nuclear magnetic resonance (NMR) analysis (26). Further analyses revealed that the cross-feeding mechanism mediated by GtfB can contribute to the pH-lowering capacity of C. albicans. Using *in situ* pH measurements via a miniature microelectrode, we found that the addition of GtfB during C. albicans biofilm formation in sucrose significantly lowered the pH values at the biofilm interface, in line with the transcriptomic-proteomic data. The foregoing acidic milieu generated by metabolic pathways of C. albicans may explain at least in part the contribution by the fungal counterpart to lowering the pH of the mixed-species biofilm microenvironment.

The combination of transcriptomics and proteomics also revealed additional mechanisms that could explain C. albicans accumulation and bacterial-fungal interactions in the mixed-species biofilm. Genes and proteins associated with hyphal growth in C. albicans were upregulated in mixed biofilms, particularly the heat shock protein (HSP) (see Table S1 in the supplemental material) (33–36). HSP is an important factor for oxidative stress responses, which may contribute to the yeast-hypha morphogenesis (37, 38). In addition, genes associated with fungal vacuolar development were upregulated (Table S1), which can modulate hyphal branching and division (39, 40). These observations help explain the presence of hyphal forms of C. albicans in the structure of the cariogenic biofilms formed *in vitro* and *in vivo* (17).

Intriguingly, genes associated with C. albicans mannan and glucan production were significantly upregulated in the presence of S. mutans. These findings are relevant to this cross-kingdom interaction because the cell wall mannans of C. albicans have been recently identified as key binding sites for GtfB (22). The GtfB bound on the fungal cell wall is enzymatically active, promoting not only coadhesion with S. mutans and mixed-biofilm development (26) but also sucrose breakdown to provide readily metabolizable sugars for C. albicans, as demonstrated here. Furthermore, β-glucans produced by C. albicans are an integral component of the extracellular matrix, contributing (together with α-glucans) to scaffolding and diffusion-limiting properties that confers stability and antifungal drug tolerance to the cross-kingdom biofilm (17, 41).

The present study also demonstrates the importance of proteomics, in addition to transcriptomics, as a powerful tool to study the *Candida* contribution and bacterial-fungal interactions in cross-kingdom biofilms. It allows access to a large and ever-improving fungal and bacterial database that can be converted to functional information using stringent analysis techniques, providing a more holistic understanding of complex biological systems such as in mixed-species biofilms. Proteomics also act as an additional platform to confirm and validate data from other omics technologies, such as transcriptomics and metabolomics. Here, we developed the initial protocols to study cross-kingdom proteomics to help understand the influences and interactions of each species in a fungal-bacterial mixed biofilm. However, we recognize the technical challenges and limitations of quantitative proteomics experiments, as inefficient chemical labeling which can compromise protein coverage may occur, in addition to sample loss due to extensive purification steps and chemical side reactions. There is also a possibility of underrepresentation of low-abundance proteins, while current analytical software and bioinformatics are not standardized across all the different omics-based platforms, which can affect data interpretation. Nevertheless, we hope this can be a first step toward developing detailed multi-omics protocols tailored to elucidate the mechanisms involved in this and other cross-kingdom polymicrobial biofilms.

**FIG 10:**
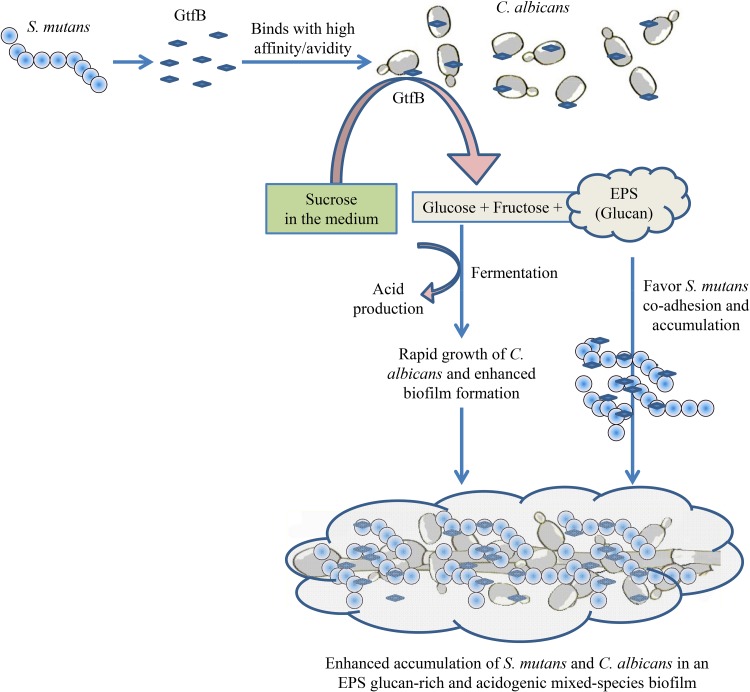
Schematic diagram showing the role of GtfB in the S. mutans-C. albicans mixed-species biofilm. GtfB attached to the C. albicans cells converts sucrose into monosaccharides and EPS glucans. The monosaccharides can be efficiently utilized by C. albicans, resulting in rapid growth of the fungus in S. mutans-C. albicans mixed-species biofilm. Monosaccharide utilization by C. albicans can also contribute with acidification and lowering the pH of the biofilm microenvironment. In parallel, glucans also promote both bacterial and fungal accumulation. The increase in C. albicans cell accumulation helps accrue more S. mutans cells into the biofilm biomass via enhanced sugar metabolism and glucan production. Taken together, the bacterial-fungal interactions and cross-feeding mediated by GtfB contribute to the microbial accumulation, reduction of pH, and generation of an acidic milieu, leading to a highly virulent (cariogenic) mixed-kingdom biofilm.

In summary, the present study used iTRAQ-based quantitative proteomics combined with RNA-Seq to comprehensively study the role of C. albicans and its interactions with S. mutans within mixed biofilms. The analysis provided new insights showing that C. albicans benefits from a symbiotic bacterial-fungal sugar metabolism and actively modulates the biofilm virulence properties under cariogenic conditions. This relationship also influences C. albicans morphogenesis and cell wall properties that can impact the biofilm structural organization and EPS-matrix assembly. Importantly, we discovered an additional functional role for GtfB in cross-feeding interactions by converting sucrose into monosaccharides which can be readily utilized by C. albicans. The foregoing factors can contribute to the growth and metabolic activity of the microorganisms as well as EPS production, facilitating the assembly of a cariogenic cross-kingdom biofilm and the generation of an augmented acidic milieu (Fig. 10) associated with severe childhood caries. Further research is warranted to understand the mechanisms that regulate the metabolic pathways, including *in situ* analysis of the biochemical reactions and sugar transport into C. albicans. In addition, time course analysis of key metabolites, carbohydrates, and specific transcripts/proteins identified here during biofilm development will be performed in follow-up studies. Ultimately, the data generated will form the basis for a longitudinal multi-omics analysis using clinical samples that could further elucidate the impact of this bacterial-fungal interaction in humans and possibly lead to new approaches to diagnose and treat a highly prevalent pediatric disease.

## MATERIALS AND METHODS

### Microbial strains and culture conditions.

C. albicans strain SC5314 and the *bcr1*Δ/Δ mutant (*ura3*::*imm434*/*ura3*::*imm434his1*::*hisG/his1*::*hisGarg4*::*hisG/arg4*::*hisGbcr1*Δ::*HIS1*/*bcr1*Δ::*UFP*) and S. mutans strain UA159 were cultured under conditions described previously (20). Briefly, C. albicans and S. mutans strains were stored at −80°C in glucose minimal medium (GMM) and tryptic soy broth (TSB), respectively, containing 50% glycerol. C. albicans was streaked on GMM agar plates, while S. mutans was streaked on brain heart infusion (BHI) agar plates.

### *In vitro* biofilm development.

Biofilms were formed by a previously established protocol with slight modifications (20). Prior to biofilm formation, C. albicans and S. mutans cells were grown to mid-exponential phase in broth culture using ultrafiltered tryptone yeast extract medium (UFTYE) (pH 5.5 and 7.0 for C. albicans and S. mutans, respectively) containing 1% (wt/vol) glucose and harvested by centrifugation (6,000 × *g*, 10 min, 4°C) as described previously (21). The cells were then washed and resuspended in phosphate-buffered saline (PBS). For the C. albicans single-species biofilm formation, the optical density (OD) of the yeast cell suspension was adjusted to a 0.375 McFarland standard (equivalent to approximately 1 × 10^7^ CFU/ml) in PBS. For mixed-species biofilm formation, a C. albicans yeast cell suspension of a 0.375 McFarland standard (equivalent to approximately 1 × 10^7^ CFU/ml) and an S. mutans cell suspension of a 0.300 McFarland standard (equivalent to approximately 1 × 10^7^ CFU/ml) were used. The cell suspensions were centrifuged, and the resulting cell pellets were resuspended in a similar volume (to the final OD-adjusted PBS volume) of fresh tryptone-yeast extract (UFTYE) broth containing 1% (wt/vol) sucrose. S. mutans and C. albicans single-species biofilms were prepared by combining half a volume of the respective OD-adjusted cultures with half a volume of fresh UFTYE–1% sucrose medium. Mixed-species biofilms were prepared by combining half a volume from both S. mutans and C. albicans OD-adjusted cultures. The biofilm formation was carried out in presterilized flat-bottom 96-well microtiter plates or 24-well microtiter plates (Greiner Bio-one). The plates were incubated at 37°C for 24 h in a 5% CO_2_ incubator with shaking set at 80 rpm.

### RNA preparation and processing.

The cells were harvested from the single- and mixed-species biofilms grown in 24-well plates, and the RNA was extracted using a RiboPure yeast RNA extraction kit (Invitrogen) according to the manufacturer’s instructions. The contaminant DNA in the samples was removed using a Ambion DNA-free kit (Invitrogen). RNA degradation and contamination were monitored on 1% agarose gels. RNA purity was checked using a NanoPhotometer spectrophotometer (Implen, CA, USA). The RNA concentration was measured using the Qubit RNA assay kit in a Qubit 2.0 fluorometer (Life Technologies, CA, USA). RNA integrity was assessed using the RNA Nano 6000 assay kit of the Bioanalyzer 2100 system (Agilent Technologies, CA, USA).

### Library preparation for transcriptome sequencing.

A total amount of 3 μg RNA per sample was used as input material for the RNA sample preparations. Sequencing libraries were generated using the NEBNext Ultra RNA Library Prep kit for Illumina (NEB, USA) following the manufacturer’s recommendations, and index codes were added to attribute sequences to each sample. Briefly, mRNA was purified from total RNA using poly(T) oligonucleotide-attached magnetic beads. Fragmentation was carried out using divalent cations under elevated temperatures in NEBNext first-strand synthesis reaction buffer. First-strand cDNA was synthesized using random hexamer primers and Moloney murine leukemia virus (M-MuLV) reverse transcriptase (RNase H). Second-strand cDNA synthesis was subsequently performed using DNA polymerase I and RNase H. In the reaction buffer, deoxynucleoside triphosphates (dNTPs) with dTTP were replaced by dUTP. Remaining overhangs were converted into blunt ends via exonuclease/polymerase activities. After adenylation of 3′ ends of DNA fragments, NEBNext adaptor with hairpin loop structure was ligated to prepare for hybridization. In order to select cDNA fragments preferentially of 150 to 200 bp in length, the library fragments were purified with the AMPure XP system (Beckman Coulter, Beverly, USA), and then 3 μl User Enzyme (NEB, USA) was used with size-selected, adaptor-ligated cDNA at 37°C for 15 min, followed by 5 min at 95°C, before PCR. PCR was then performed with Phusion high-fidelity DNA polymerase, universal PCR primers and Index (X) primer. Finally, PCR products were purified (AMPure XP system), and library quality was assessed on the Agilent Bioanalyzer 2100 system.

### Sequencing and mapping reads to reference genome.

Cluster generation of the index-coded samples was performed on a cBot cluster generation system using the HiSeq PE cluster kit cBot-HS (Illumina) and sequenced on an Illumina HiSeq platform, and 125-bp/150-bp paired-end reads were generated. Raw data (raw reads) of fastq format were processed through in-house Perl scripts. Reference genome and gene model annotation files were downloaded from genome websites directly. For C. albicans, the EnsemblFungi database was used, and for S. mutans, the EnsemblBacteria database was used. For the prokaryotic S. mutans, building the index of the reference genome and aligning clean reads to the reference genome were performed using Bowtie 2.2.3. For the eukaryotic C. albicans, the index of the reference genome was built using Bowtie 2.2.3, and paired-end clean reads were aligned to the reference genome using TopHat v2.0.12. TopHat was selected as the mapping tool because TopHat can generate a database of splice junctions based on the gene model annotation file and thus has a better mapping result than other, nonsplice mapping tools.

### Quantification and differential gene expression analysis.

HTSeq v0.6.1 was used to count the number of sequencing reads mapped to each gene. Thereafter, the fragments per kilobase per million (FPKM) for each gene was calculated based on the length of the gene and read count mapped to the gene. FPKM is the expected number of fragments per kilobase of transcript sequence per million base pairs sequenced, which considers the effect of sequencing depth and gene length for the read count at the same time. This is currently the most commonly used method for estimating gene expression levels (42). Differential expression analysis was performed using the DESeq R package (1.18.0). Another level of cutoff was applied by selecting genes with a log_2_ fold change of 1, which was considered the threshold for significantly differential expression.

### GO pathway analysis and KEGG enrichment analysis of differentially expressed genes.

The “gene IDs” of the identified upregulated and downregulated genes were mapped to the “protein IDs” in the UniProt database, and these proteins were subjected to gene ontology (GO) analysis using Cytoscape (v2.8.3) (43) with the BINGO plugin (v2.44) (44). The figures generated were abstracted from Cytoscape as previously described (45). Statistical enrichment of differentially expressed genes in KEGG pathways (http://www.genome.jp/kegg/) was analyzed using the KOBAS software.

### Protein extraction and preparation for iTRAQ analysis.

S. mutans and C. albicans single-species and mixed-species biofilms grown in 24-well microtiter plates (Greiner Bio-one) for 24 h were used for the protein extraction. Biofilms were washed once with PBS, and cell pellets were solubilized in triethylammonium bicarbonate (TEAB) buffer. Samples were homogenized using glass beads (0.5 mm) in an Omni Bead Ruptor 24 (Omni International Inc.) following operator protocol, and then 1% SDS was added and heated at 60°C for 10 min. Lysates were collected, and the protein concentration was determined using the RC-DC assay (Bio-Rad). Initial protein estimation showed that the mixed-species biofilm contains a 1:3 ratio (25% to 75%) of S. mutans and C. albicans proteins (see Fig. S4 in the supplemental material). Proteins extracted from single-species S. mutans and C. albicans biofilms were appropriately pooled to form a similar ratio for comparison against the mixed-species protein sample. For each sample, 100 μg of proteins was taken for downstream proteomics experiments. The sample was polymerized in a gel containing 4% SDS and subsequently fixed with a fixing reagent (50% methanol, 12% acetic acid) for 30 min at room temperature. The gel was cut into small pieces (1 mm^3^). The finely cut pieces of gel were washed with 50 mM TEAB–50% (vol/vol) acetonitrile and dehydrated using 100% acetonitrile, and the step was repeated three times. Subsequently, samples were reduced with 5 mM TCEP at 57°C for 60 min, followed by alkylation with 10 mM methyl methanethiosulfonate (MMTS) for 60 min at room temperature with occasional vortexing. Following reduction and alkylation, the gel pieces were washed with 500 μl of 50 mM TEAB, the gel pieces were dehydrated by adding 500 μl of acetonitrile, and then 500 μl of 50 mM TEAB was added for reswelling. A final dehydration step was performed using 100 μl of acetonitrile, 1 μg of trypsin per 20 μg of proteins was added, and trypsinization was performed at 37°C for 16 h. The digested peptides were extracted sequentially with 200 μl each of 50 mM TEAB, 5% formic acid in 50% acetonitrile, and 100% acetonitrile. The solutions were added, allowed to stand for 5 to 10 min, and centrifuged at 6,000 rpm. The supernatants with the digested peptides were collected, combined, and stored at –20°C before liquid chromatography (LC) separation and mass spectrometry (MS) analysis. The samples were lyophilized until the tubes were completely dried, and 30 μl of the dissolution buffer was added. The protein digests were then labeled with the iTRAQ Reagents 8-plex kit (Sciex, Foster City, CA) following the manufacturer’s protocol. Isobaric tags 113 and 114 were used to tag two samples/replicates containing pooled proteins from S. mutans and C. albicans single-species biofilms (according to a 1:3 ratio as described above). Isobaric tags 115, 116, 117, 118, 119, and 121 were used to tag six samples/replicates of proteins from mixed-species biofilms. The eluates were then desalted in a Sep-Pak C_18_ cartridge (Waters, Milford, MA), dried, and then reconstituted in 20 mM ammonium formate in water at pH 10 before two-dimensional (2D) LC-tandem MS (LC-MS/MS) analysis was performed.

### 2D LC-MS/MS analysis.

The 2D LC-MS/MS analysis was performed according to a methodology previously described by our group (46, 47). The first dimension of the peptide separation was performed using a 1290 Infinity LC system (Agilent) linked with a reversed-phase column. The micropickup loop mode injected approximately 8 μg of the labeled peptide mixture into the Xbridge C_18_ column (3.5 μm, 3.0 mm by 150 mm; Waters Corp., Milford, MA). Mobile phase A consisted of 20 mM ammonium formate in water (pH 10), and mobile phase B consisted of 20 mM ammonium formate in 80% acetonitrile (pH 10). A total of 96 elute fractions were collected on a 96-well V-bottom plate at a flow rate of 0.2 ml/min. The step gradients of mobile phase B were set as follows: 0% for 5 min, 0 to 15% for 15 min, 15 to 40% for 40 min, 40 to 80% for 1 min, hold at 80% for 5 min, 80 to 0% for 1 min, and continuation at 0% for another 17 min. The eluted fractions were combined into nine fractions, lyophilized, and reconstituted in 98% water, 2% acetonitrile, and 0.1% formic acid. The second dimension of peptide separation was conducted using an Eksigent nanoLC Ultra and ChiPLC-nanoflex (Eksigent, Dublin, CA) in TrapElute configuration. Subsequently, the samples were loaded on a column (200 μm by 0.5 mm) and eluted on an analytical column (ChromXP C18-CL; 75 μm by 15 cm, 3 μm). A gradient formed by mobile phase A (2% acetonitrile, 0.1% formic acid) and mobile phase B (98% acetonitrile, 0.1% formic acid) was used to separate 2 μl of the sample at a 0.3-μl/min flow rate. The following gradient elution of mobile phase B was used for peptide separation: 0% to 5% for 1 min, 5% to 12% for min, 12% to 30% for 104 min, 30% to 90% for 2 min, 90% to 90% for 7 min, 90% to 5% for 3 min, and finally a hold at 5% for 13 min. The tandem MS analysis was performed using a 5600 TripleTOF system (Sciex) in information-dependent mode. The mass range of 400 to 1800 *m/z* and accumulation times of 250 ms per spectrum were chosen for precursor ion selection. MS/MS analysis was performed on the 20 most abundant precursors (accumulation time, 100 ms) per cycle with 15-s dynamic exclusion. MS/MS was done in high-sensitivity mode with rolling collision energy.

### Protein identification and quantification.

Peptide identification and quantification were carried out with ProteinPilot 5.0 software revision 4769 (AB Sciex) using the Paragon database search algorithm (5.0.0.0.4767) and the integrated false-discovery rate (FDR) analysis function. The data were searched against two protein sequence databases downloaded from UniProtKB for C. albicans SC5314 on 25 June 2017 (total of 12,080 entries) and for S. mutans UA159 on 25 June 2017 (total of 3,918 entries). The MS/MS spectra obtained were searched using the following user-defined search parameters: sample type, iTRAQ 8-plex (peptide labeled); cysteine alkylation, MMTS; digestion, trypsin; instrument, TripleTOF5600; special factors, none; species, Candida albicans and Streptococcus mutans; ID focus, biological modification; database for C. albicans, uniprot_170626_Kasspa_CA.fasta; database for S. mutans, uniprot_170626_Kasspa_SA.fasta; search effort, thorough; and FDR analysis, yes. The MS/MS spectra were searched against a decoy database to estimate the FDR for peptide identification. The decoy database consisted of reversed protein sequences from the C. albicans database and S. mutans database. FDRs for protein and peptides were estimated as 1.7% and 2.7%, respectively. The resulting data set was auto-bias corrected to remove any variations imparted because of the unequal mixing during the combination of different labeled samples. Different modification states of the same peptide sequences are considered distinct by the software.

### Determining the fold cutoff for identifying upregulated and downregulated proteins.

To identify the proteins using ProteinPilot 5.0 software, a strict unused score cutoff of ≥1.3 was adopted as the qualification criterion to ensure that the proteins were identified with a ≥95% peptide confidence level. The removal of proteins which did not qualify in this step resulted in 1,718 C. albicans proteins and 686 S. mutans proteins. Among the proteins that satisfied this stringent cutoff, only proteins which were identified by ≥2 peptides were selected for further analysis. After this level of filtration, 1,384 C. albicans proteins and 530 S. mutans proteins which had two or more peptides were identified. These proteins were then filtered using a population statistics-based methodology to determine the cutoff for proteins with abundance changes (48). In order to obtain a proteomic expression data set, the comparison was performed between two replicates of pooled proteins from S. mutans and C. albicans single-species biofilms (tagged with isobaric tags 113 and 114) and six protein samples/replicates from mixed-species biofilms (tagged with isobaric tags 115, 116, 117, 118, 119, and 121) (see Fig. S1A in the supplemental material). The expression level of a protein was determined by comparing the average of 12 ratios ([i] ratio 1, 115:113; ratio 2, 116:113; ratio 3, 117:113; ratio 4, 118:113; ratio 5, 119:113; and ratio 6, 121:113; [ii] ratio 1, 115:114; ratio 2, 116:114; ratio 3, 117:114; ratio 4, 118:114; ratio 5, 119:114; and ratio 6, 121:114) (Fig. S1B). The average of each of the 12 ratios was transformed to log_2_ values. These 12 sets of log_2_ ratios were compared with corresponding sets of 12 log_2_(0) dummy ratios representing no change in the protein abundance. The comparison was performed using the one-sample *t* test since the data were normally distributed. Proteins showing *P* values of *<*0.05 for the mean iTRAQ ratios were considered statistically significant and selected for further analysis.

The percent variations of proteins of C. albicans and S. mutans with altered abundance were then plotted against the cumulative percent coverage (see Fig. S5 in the supplemental material), and the variation against 88% coverage was taken into account to determine the fold cutoff, considering the population outside 88% significantly altered. When this method was applied to both the data sets, we observed about 37% variation for the C. albicans data set and 31% variation for the S. mutans data set, corresponding to 88% coverage of data. Based on the above calculation, the cutoff was fixed at 1.37-fold (37% variation), corresponding to the iTRAQ ratios of >1.37 for increased abundance and <0.729927 (1/1.37) for decreased abundance for the C. albicans data set. Similarly, for the S. mutans data set, the cutoff for increased abundance was fixed as >1.312, and the cutoff for decreased abundance was fixed as <0.762195 (1/1.312). The aforementioned cutoff values were then applied to the average of the 12 replicates of both data sets. Another level of filtering was used by applying the Student *t* test to the proteins, where abundance change was considered significant when the *P* value was less than 0.05 between samples. Proteins which were similarly expressed in single-species and mixed-species biofilms were considered to have “no change.” The proteins which were differentially expressed between single-species and mixed-species biofilms were classified as having “increased abundance” or “decreased abundance.” The distribution of the protein expression of C. albicans and S. mutans mixed-species biofilms compared to that for C. albicans and S. mutans was plotted on volcano plots. Identified proteins were subjected to gene ontology analysis using Cytoscape (v2.8.3) (43) with the BINGO plugin (v2.44) (44).

### Biofilm quantification.

Biofilm quantification was performed using the XTT reduction assay and CFU counting method as we have previously described (49).

**(i) XTT reduction assay.** In brief, the culture medium was aspirated, and the biofilms were washed with 200 μl of PBS once to remove nonadhered cells. A volume of 200 μl of the XTT solution (containing 4 μM menadione and 0.2 mg/ml XTT in PBS) was introduced into the biofilms and incubated in the dark for 20 min at 37°C. The subsequent colorimetric changes in the XTT reagent were measured at 490 nm using a spectrophotometer (Multiskan GO; Thermo Scientific) after transferring the solution into a new plate.

**(ii) CFU counting method.** The cells from the biofilms were harvested from the 96-well plates using PBS, and a dilution series was prepared. Cells from each dilution was plated on GMM agar plates. The agar plates were incubated at 30°C for 48 h, and the developed fungal colonies were counted for CFU evaluation.

### Growth kinetics of C. albicans strains.

A C. albicans yeast cell suspension of a 0.375 McFarland standard (equivalent to approximately 1 × 10^7^ CFU/ml) was diluted in tryptone-yeast extract (UFTYE) broth containing 1% (wt/vol) sucrose, S. mutans supernatant, or S. mutans-C. albicans supernatant in 96-well microtiter plates (Greiner Bio-one). The optical density of the microbial cultures was measured at a wavelength of 600 nm using a spectrophotometer (Multiskan GO; Thermo Scientific). Measurements were taken at 30-min intervals up to 48 h.

### CLSM.

For confocal laser scanning microscopy (CLSM), C. albicans biofilms were grown using UFTYE broth containing 1% (wt/vol) sucrose, S. mutans supernatant, or S. mutans-C. albicans supernatant in Thermanox (Nunc) 8-well plates under experimental conditions similar to those described above. Biofilms were stained with 1% (vol/vol) calcofluor white (Sigma-Aldrich) and observed using an Olympus FluoView FV1000 confocal microscope. Z sections were collected and analyzed using the Imaris software. Estimation of the biofilm biovolumes was also performed using the Imaris software.

### Preparation of cell-free supernatants.

Biofilm supernatants of 24-h biofilms were collected from single-species S. mutans biofilms (S. mutans supernatant) and S. mutans-C. albicans mixed-species biofilms (S. mutans-C. albicans supernatant), i.e., S. mutans-C. albicans SC5314 mixed-species biofilm supernatant (S. mutans SC5314 supernatant) and S. mutans-C. albicans *bcr1*Δ/Δ mixed-species biofilm supernatant (S. mutans *bcr1*Δ/Δ supernatant). The supernatants were subjected to centrifugation for 10 min and filtered through a 0.22-μm-pore-size membrane filter (surfactant-free cellulose acetate; Sartorius) to collect the cell-free supernatant. These supernatants were subsequently used for the single-species C. albicans biofilm formation, sugar testing, and pH measurements.

### Testing for monosaccharides.

Qualitative determination of monosaccharides was performed using standard techniques. Glucose in the supernatants was tested using Benedict’s reagent (10 g NaCO_3_, 17.3 g sodium citrate, and 1.7 g CuSO_4_·5H_2_O in 95 ml of distilled water). A volume of 2 ml of the supernatant was added to 1 ml of Benedict’s reagent and heated at 80°C for 10 min in a water bath. A positive result showed a brown coloration of the supernatant. Fructose in the supernatant was detected using Seliwanoff’s reagent (0.1 g of resorcinol in 100 ml of 4 M HCl). A volume of 1 ml of the sample was added to 2 ml of Seliwanoff’s reagent and heated at 80°C for 3 min. A bright red coloration indicates the presence of fructose. To determine the glucose utilization rates of S. mutans and C. albicans strains, a commercial glucose assay kit was used (Sigma-Aldrich). The biofilm supernatant of each microorganism collected every 4 h for a time period of 24 h was filtered and used for the assay following the manufacturer’s protocol.

### pH testing.

The filtered biofilm supernatants (S. mutans single species, C. albicans single species, and S. mutans-C. albicans mixed species) collected every 4 h for 24 h were used for pH measurements with a pH electrode. Purified GtfB preparations were added to C. albicans grown in UFTYE–1% sucrose medium, and the pH of the biofilm microenvironment was measured using microelectrodes after 24 h. *In situ* pH measurements were conducted by placing the tip of a Beetrode pH electrode (World Precision Instruments, New Haven, CT, USA) into the biofilm, and pH readings were recorded as described previously (50).

### Statistical analysis.

DESeq was used for differential gene expression analysis in RNA-Seq, and *P* values were adjusted using the Benjamini-Hochberg approach for controlling the false-discovery rate. Genes with an adjusted *P* value of <0.05 were assigned as differentially expressed. In iTRAQ proteomics, a population statistics method was applied to select the cutoff for proteins with abundance changes (48). For biochemical and quantitative assays such as the XTT assay, CFU assay, biofilm biovolume analysis, pH measurements, and monosaccharide testing, experiments were performed in at least three biological replicates. Pairwise comparison and significance of the results were analyzed using the two-sample *t* test for parametric data sets and the Mann-Whitney U test for nonparametric data sets. Differences were considered significant when *P* values were <0.05. Analysis was performed using SPSS (version 16.0, SPSS Inc.).

### Accession number(s).

The mass spectrometry proteomics data have been deposited to the ProteomeXchange Consortium via the PRIDE (51) partner repository with the data set identifier PXD010261.

## Supplementary Material

Supplemental file 1

Supplemental file 2

Supplemental file 3

Supplemental file 4

Supplemental file 5
