# Supplementary material for: Multi-omics Analyses Reveal Synergistic Carbohydrate Metabolism in Streptococcus mutans-Candida albicans Mixed-Species Biofilms
Source: Infect Immun. 2019 Sep 19;87(10):e00339-19. doi: 10.1128/IAI.00339-19 (PMC6759298; doi:10.1128/IAI.00339-19)
Supplement: Supplemental file 5 [file IAI.00339-19-s0005.pdf]

Supplementary Fig 1: Schematic representation of the experimental design for iTRAQ labeling

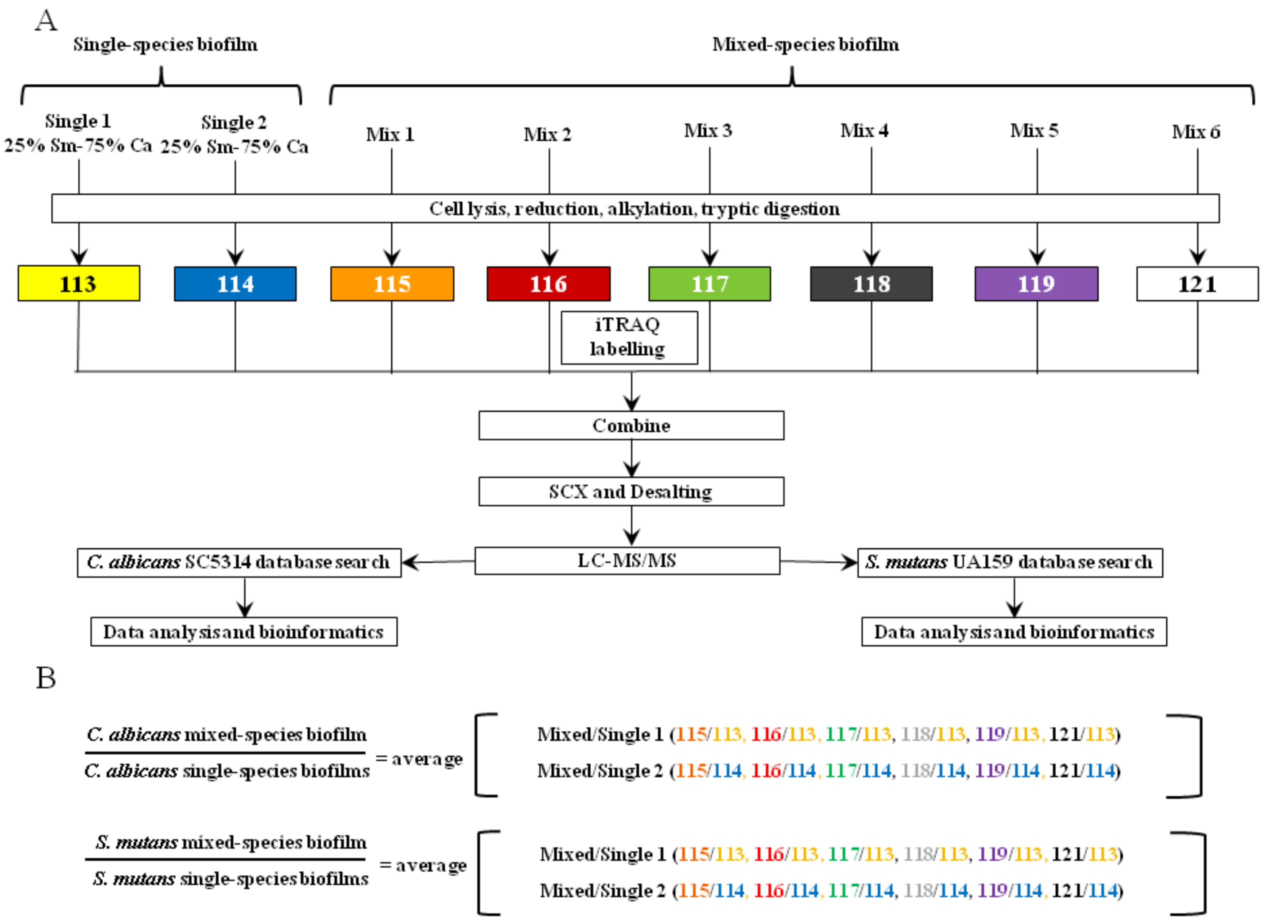

(A) Two replicates from Sm25%-Ca75% protein mix of the single-species biofilm and six replicates of the proteins from the mixed-species biofilm were labeled with iTRAQ labeling reagents 113, 114 and 115, 116, 117, 118, 119, 121 respectively. (B) Protein expression of each species in mixed-species biofilm compared with respective single-species biofilms was determined by taking the average of all cross comparisons between each replicate of the mixed-species biofilm vs single-species biofilm protein mix.

Supplementary Fig 2. Flow chart illustrating the different filtering methodology used to analyze the abundance changed proteins in *S. mutans* mixed-species biofilms and single-species biofilms

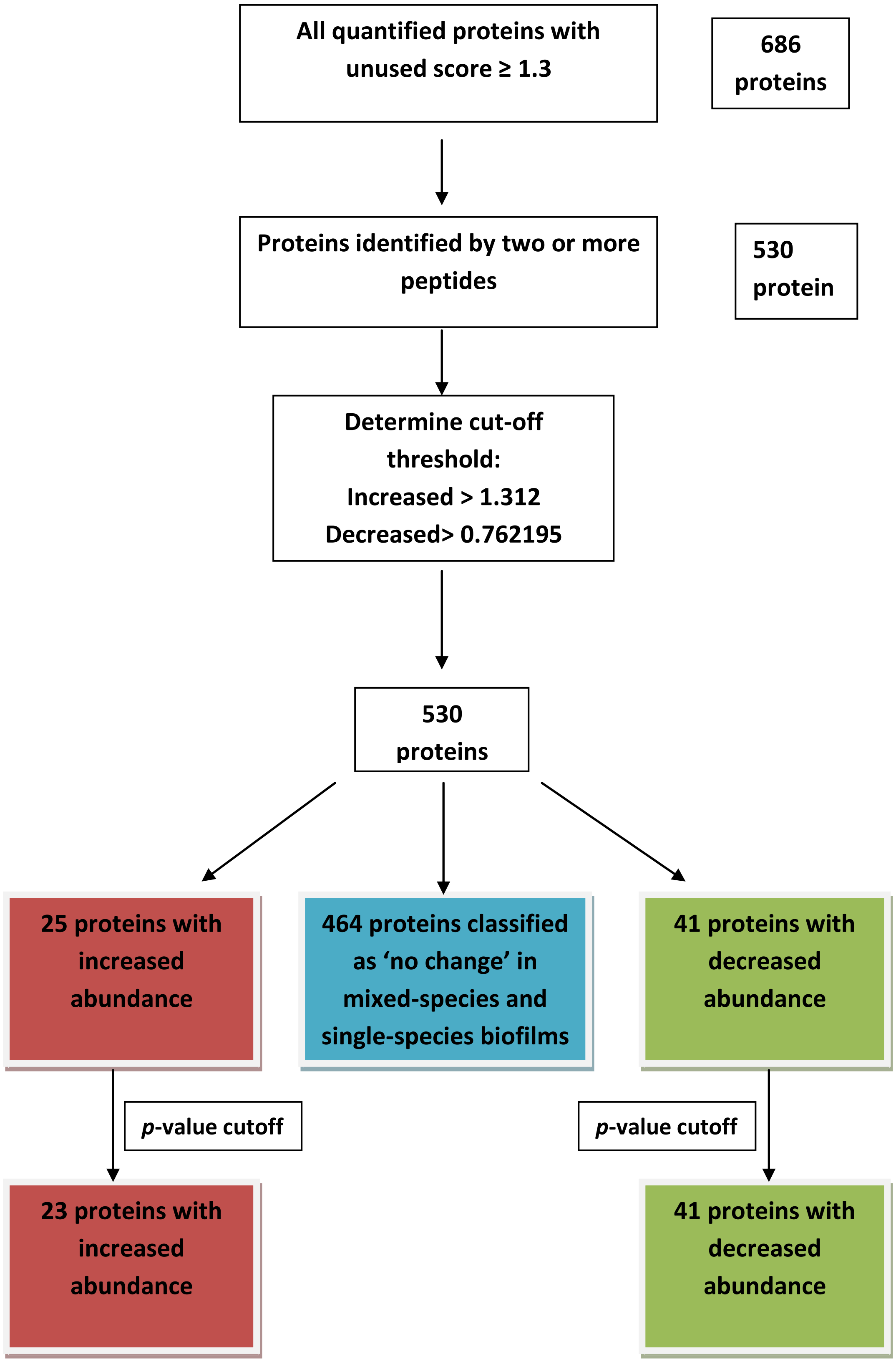

Supplementary Fig 3. Flow chart illustrating the different filtering methodology used to analyze the abundance changed proteins in *C. albicans* mixed-species biofilms and single-species biofilms

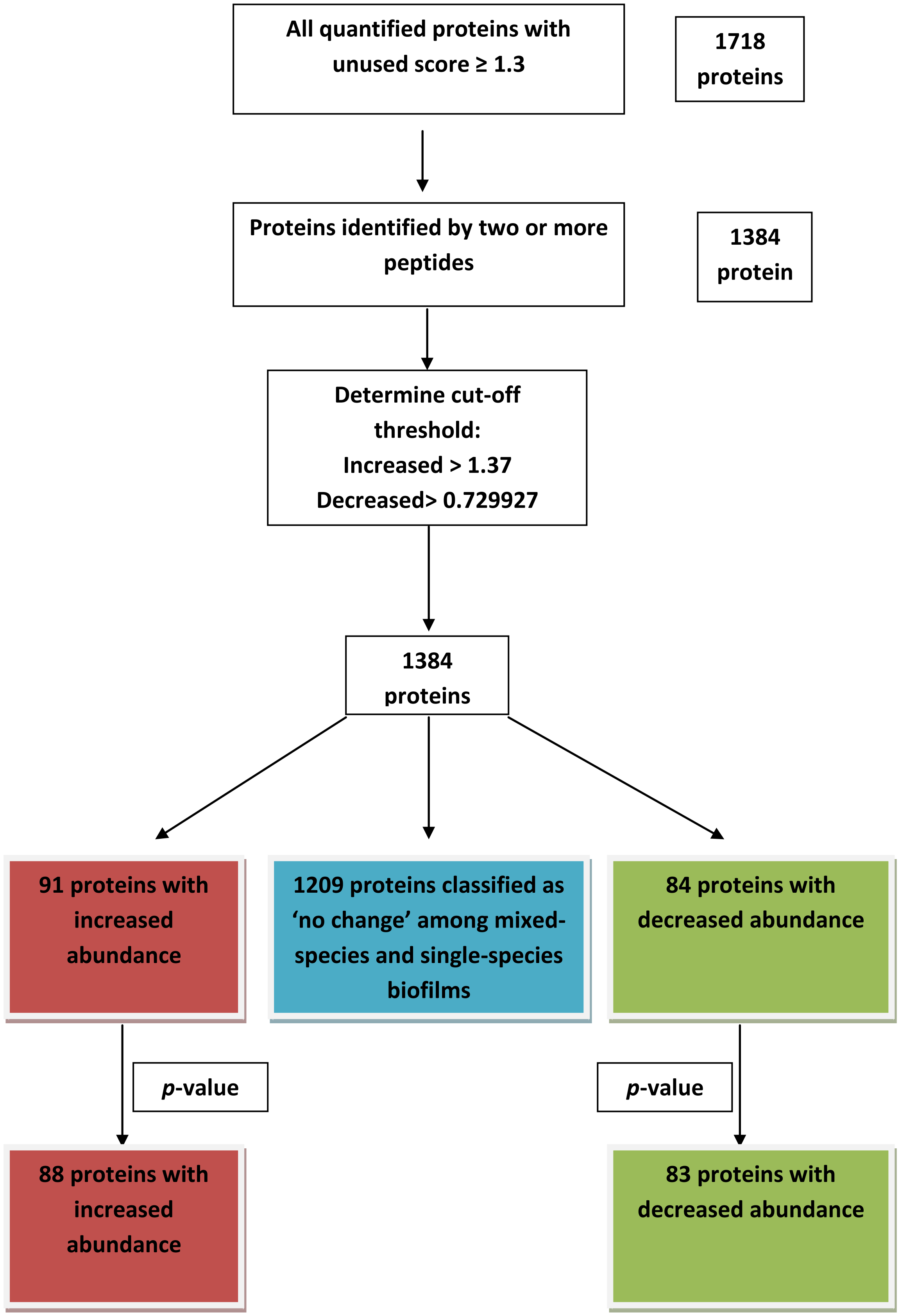

Supplementary Fig 4: Normalization of proteins in the mixed-species and single-species biofilms

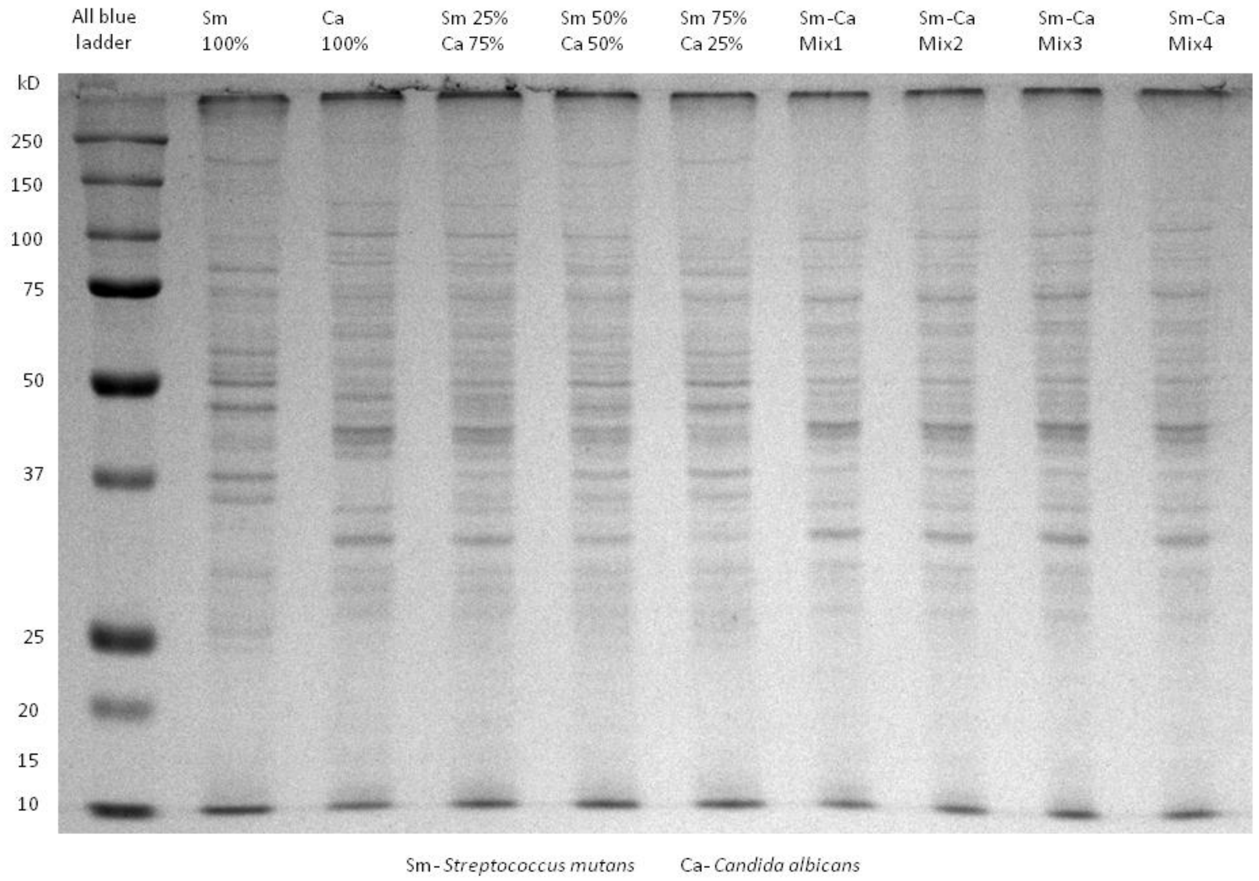

The mixed-species biofilm had different amount of proteins derived from *S. mutans* and *C. albicans* counterparts. We compared the amount of proteins from each organism in the mixed-species biofilm (Sm-Ca Mix 1,2,3,4) against the protein amounts derived from *S. mutans* and *C. albicans* single-species biofilms, mixed according to specific ratios (Sm100%-Ca0%; Sm0%-Ca100%; Sm25%-Ca75%; Sm50%-Ca50%; Sm75%-Ca25%). We identified that a protein mixture of 25% of *S. mutans* proteins and 75% of *C. albicans* proteins single-species biofilms mixed together corresponds to the amount of *S. mutans* and *C. albicans* proteins detected in the mixed-species biofilm.

Supplementary Fig 5. Percentage variation in iTRAQ ratios of the same proteins found in various replicates. The primary vertical axis represents the corresponding number of proteins (bars) having different % co-efficient of variation (%CV) that was plotted in the horizontal axis. The secondary vertical axis represents the cumulative % of the counted proteins (lines). Variation against 88% coverage was taken into account for determining the fold cut-off, considering the population outside 88% as significantly altered.

I. ***C. albicans*** **-** 37% variation corresponding to >1.37 for increased abundance and <0.729927 (1/1.37) for decreased abundance

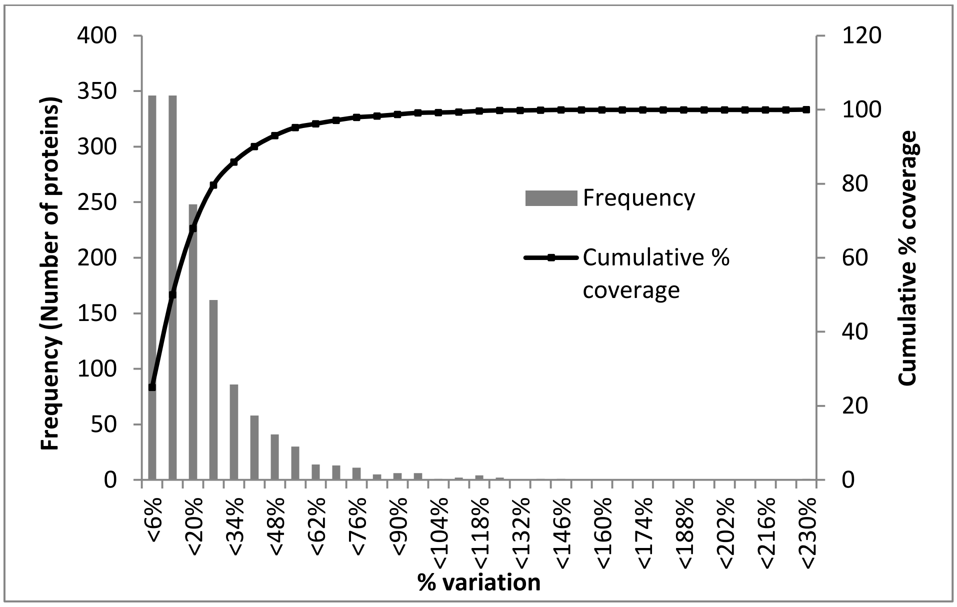

II. ***S. mutans*** - 31% variation corresponding to >1.312 for increased abundance and <0.762195 (1/1.312) for decreased abundance

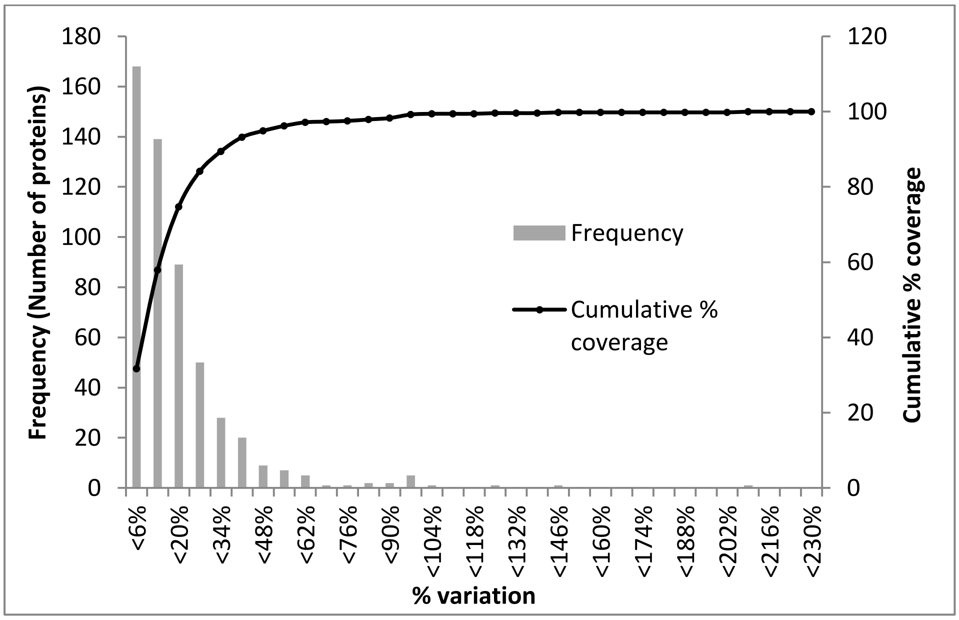

Supplementary table 1: Some important *Candida albicans* genes affected in Sm-Ca mixed-species biofilms

1. **Genes involved in hyphal formation**

I. Genes directly involved in hyphal formation

gene/genes	function	reference
CEK2 (*W5Q_06054)*	encoding a MAPK protein important for filamentous growth	
BNI1 (W5Q_01255)	required for the maintenance of polarized hyphal growth was upregulated. *BNI1*-mediated actin cables are necessary for positioning the golgi complex to a putative site of germ tube emergence and for coordinating the transport and deposition of membrane and cell wall material to a growing hypha. A study using *in vivo* time-lapse microscopy showed that deletion of the *C. albicans BNI1* results in polarity defects during yeast growth and hyphal stages	(1), *
SHO1 (W5Q_00881)	a gene involved in invasive filamentation into semi-solid medium that plays a role in morphological dimorphic transition was upregulated	*
FGR27 (W5Q_01867), *SUV3* (W5Q_03522), *HSP21* (W5Q_01833), *SLN1* (W5Q_05559), *CAS5* (W5Q_03421), *SSU1* (W5Q_06368), *TPS1* (W5Q_06032)	involved in hypha formation	*
TPS1 (W5Q_06032)	encoding an α,α-trehalose-phosphate synthase was upregulated. Disruption of the *C. albicans TPS1* gene has shown to impair the formation of hyphae and decreased infectivity	(2),*

II. Genes involved in transcriptional regulation related to the filamentous growth of the fungi

FKH2 (W5Q_02558)	a potential forkhead-like transcription factor similar to *S. cerevisiae FKH2* (YNL068C) involved in pseudohyphal growth. A study showed that Fkh2 during hyphal growth regulates *C. albicans* pathogenesis	(3), *
ADR1 (W5Q_03549)	upregulated gene that encodes transcriptional regulation of hyphal growth	*
EFG1 (W5Q_04510)	the key transcription factors in *C. albicans* involved in the yeast to hypha transition	*
RFG1 (W5Q_05709)	encodes a transcription regulator that functions in both the positive and negative regulation of filamentous growth, depending upon the environmental conditions. Rfg1p regulates genes encoding cell wall components that are specifically expressed in the filamentous forms such as *HWP1*, *RBT1*, *HYR1*, *ECE1*, *ALS1*, *RBT4* and *RBT5*	*
FGR23 (W5Q_02736) and *FGR27* (W5Q_03522)	encoding transcription factors involved in filamentous growth was upregulated	*

III. Other genes indirectly involved in hyphal growth

VPS34 (W5Q_00648)	a gene mainly involved in vacuolar sorting and segregation encoding a Phosphatidylinositol 3-kinase in *C. albicans* which has been shown to be important for filamentous growth was upregulated. Expression studies have indicated that the vps34 mutant reacts to the phenotypical defects with an up-regulation of genes involved in filament formation such as *ALS1* and *HWP1*	(4), *
MYO2 (W5Q_01331)	encoding myosin, a protein that modulate the structure of the actin cytoskeleton shown to be essential for polarized growth and hyphal transition	(5), *
SIR2 (W5Q_01578)	shown to be involved in the phenotypic switching	(6), *
MDS3 (W5Q_03241)	identified as a new member of the TOR pathway that contributes to the morphogenesis in *C. albicans*	(7), *
ECM7 (W5Q_03320)	involved in cell wall maintenance, oxidative stress response and hyphal development	(8), *
HWP2 (W5Q_03621)	encoding Hwp2p, a cell wall GPI-anchored cell wall protein that was previously shown to be necessary for hyphal and invasive growth on solid media was upregulated	(9), *

2. Genes associated with fungal vacuolar development genes were upregulated

VPS8 (W5Q_02560) and *VPS13* (W5Q_03896)	proteins associated with vacuolar development were upregulated	*
VPS15 (W5Q_04709)	a protein kinase involved in vacuolar protein sorting	*
VAC7 (W5Q_03740)	encoding a vacuolar segregation protein were also upregulated	*
AVT42 (W5Q_03055)	a potential transmembrane amino acid transporter that controls efflux of large neutral amino acids in vacuolar-like organelles	*

3. Genes associated with biofilm dispersal was upregulated in *Candida albicans*

SET3 (W5Q_01366) and *SNT1* (W5Q_01183)	genes were upregulated which are two parts of the four core members of a conserved histone deacetylase complex (Set3, Hos2, Snt1, and Sif2). Histone deacetylase complex members have been shown to play a vital role in biofilm formation, including dispersal of cells from biofilms	(10), *
YWP1 (W5Q_02272)	encoding a potential yeast form cell wall protein was upregulated, which plays an anti-adhesive role and promotes dispersal of yeast forms allowing the organism to seek new sites for colonization.	*
DIT1 (W5Q_05894)	required for biosynthesis of dityrosine in the outer layer of the spore wall	*
DTR1 (W5Q_05892)	encoding a dityrosine transporter essential for spore wall maturation was among the upregulated genes	*

4. *Candida albicans* genes in cation uptake and transport were upregulated

FTR1 (W5Q_01365)	encoding one of two plasma membrane iron permeases was upregulated. Microbial pathogens must compete with the iron-withholding defense systems of their host to acquire this essential nutrient. A study showed that out of two high-affinity iron permease genes *FTR1* and *FTR2*, *FTR1* expression was induced under iron-limited conditions and repressed when iron supply was sufficient	(11), *
FET3 (W5Q_04636)	was upregulated encoding an iron transport multicopper ferroxidase required for Fe^{2+} high affinity uptake, which oxidizes Fe^{2+} and release it from the transporter	*
CCH1 (W5Q_00104)	encoding a likely iron transport protein	*
ALR1 (W5Q_02751)	encoding a magnesium transporter	*
SMF12 (W5Q_03402)	encoding a manganese transporter	*

5. Genes associated with heat shock proteins were upregulated

CDC37 (W5Q_04837), *AHA1* (W5Q_06467) and *CNS1* (W5Q_00050)	encoding Hsp90 co-chaperones in *C. albicans*	*
HSP21 (W5Q_01833)	upregulated gene required for invasive growth and filament formation under various filament inducing conditions	*
HSP60 (W5Q_06099), *HSP70* (W5Q_01302), *HSP104* (W5Q_06286) and *STI1* (W5Q_04212)	Encoding heat shock proteins	*
HSP78 (W5Q_01774)	Hsp78 acts in concert with mitochondrial Hsp70, for the dissociation, resolubilization and refolding of aggregates of damaged proteins in the mitochondrial matrix after heat stress	*
HCH1 (W5Q_04765)	encodes a co-chaperone that binds to the molecular chaperone HSP82 and stimulates its ATPase activity	*

6. *Streptococcus mutans* enhances *Candida albicans* genes associated with drug transport

MTE4 (W5Q_00077)	potential Multi Antimicrobial Extrusion (MATE) family drug/sodium antiporter	*
QDR3 (W5Q_04716)	multidrug resistance transporter	*
YHK8 (W5Q_04766)	encoding a probable drug/proton antiporter YHK8	*
CDR1 (W5Q_02918)	encoding the multidrug resistance protein CDR1 shown to be resistant against cycloheximide	*
CDR3 (W5Q_03632)	which encodes the opaque-specific ABC transporter CDR3 involved in drug export	*
MDR1(W5Q_04891)	encoding a multidrug resistance protein was also upregulated	*

7. *Streptococcus mutans* enhances *Candida albicans* carbohydrate metabolism

ITR1 (W5Q_02514)	important for the acquisition of inositol	(12), *
ITR2 (W5Q_01924)	associated with potential sugar transporter activity was upregulated	*
SNF3 (W5Q_03002)	expression was enhanced encoding the Snf3p in *S. cerevisiae* analogues to the Hgt4 protein of *C. albicans* (orf19.5962) which acts as glucose sensors and govern sugar acquisition by regulating the expression of genes encoding hexose transporters	*
GAL1 (W5Q_00201)	encodes galactokinase similar to *S. cerevisiae* GAL3 associated with galactose metabolism	*
ARA1 (W5Q_02234D)	encoding arabinose dehydrogenase involved in arabinose metabolism	*
TPS3 (W5Q_02484)	encoding regulatory subunit of trehalose-6-P synthase involved in trehalose metabolism	*
GPM1 (W5Q_00421)	encoding the protein phosphoglycerate mutase which is involved in the third step of the subpathway that synthesizes pyruvate from D-glyceraldehyde 3-phosphate in glycolysis	*
FUMH (W5Q_03272)	encodes a fumarate hydratase were also upregulated in tri-carboxylic acid cycle	*
CYC2 (W5Q_04301)	encoding a cytochrome c mitochondrial import factor similar to *CYC2* in *S. cerevisiae*	*
COX15 (W5Q_04661)	encodes cytochrome c oxidase assembly protein COX15 involved in biosynthesis of heme A during cellular respiration	*
PDP1 (W5Q_06274)	encodes a pyruvate dehydrogenase phosphatase	*
PDC2 (W5Q_00962)	essential for the synthesis of pyruvate decarboxylase and encodes a transcriptional regulator for the pyruvate decarboxylase gene	*
YAT1 (W5Q_00164)	contributes to the transport of acetyl-CoA from the cytosol during growth on ethanol or acetate	*
LPG20 (W5Q_00403)	encoding a putative aryl alcohol dehydrogenase	*
SAD3 (W5Q_01879)	encoding one of a tandem pair of alcohol dehydrogenase	*
ADH2 (W5Q_00801)	encoding an alcohol dehydrogenase 2 which catalyzes the conversion of ethanol to acetaldehyde	*
CYB2 (W5Q_01316)	encoding Cyb2, a heme-containing dehydrogenase (L-lactate cytochrome-*C* oxidoreductase) essential for the utilization of L-lactate as a carbon source	*
DLD1 (W5Q_01734)	a putative D-lactate dehydrogenase	*

8. *Streptococcus mutans* enhances peroxisomal assembly and fatty acid oxidation in *Candida albicans*

PEX1 (W5Q_05290), *PEX6* (W5Q_01973), *PEX11* (W5Q_05007 and W5Q_05034) and *PEX29* (W5Q_04167)	encoding peroxisomal membrane proteins	*
FOX2 (W5Q_02570)	involved in fatty acid β-oxidation. A study showed that a *fox2Δ/fox2Δ* mutant, displayed strong growth defects on nonfermentable carbon sources other than fatty acids (e.g., acetate, ethanol or lactate)	(13), *
SCT1 (W5Q_02571), *POT2* (W5Q_02635), *POX102* (W5Q_02683), *POX104* (W5Q_02686), *FAA21* (W5Q_02795) and *ADR1* (W5Q_03549)	genes involved with peroxisomal fatty acid beta-oxidation	
MLS1 (W5Q_00935)	encoding malate synthase 1, a key enzyme in the glyoxalate pathway	*
SAK1 (W5Q_03749)	SNF1-activating kinase 1 protein in the glyoxalate cycle. A study using sak1 mutants showed that Sak1p ensures basal expression of glyoxylate cycle and gluconeogenesis genes even in glucose-rich media and thereby contributes to the metabolic plasticity of *C. albicans*	(14), *

9. *Streptococcus mutans* alters *Candida albicans* cell wall and cell membrane properties

I. Mannan production

MNN1 (W5Q_04290)	encoding a putative alpha-1,3-mannosyltransferase	*
MNN11 (W5Q_00231)	encoding alpha-1,6-mannosyltransferase	*
MNN26 (W5Q_05390)	encoding alpha-1,2-mannosyltransferase	*
ALG11 (W5Q_04805)	encoding an alpha-1,2-mannosyltransferase	*
BMT7 (W5Q_02844)	encoding beta-mannosyltransferase	*
MNN4 (W5Q_04865), *MNN42* (W5Q_01801) and *MNN41* (W5Q_01803)	involved in transfer of mannosylphosphate to cell wall mannans were similarly upregulated	*
WRY7 (W5Q_00489)	encoding a putative beta-mannosyltransferase required for the addition of beta-mannose to the acid-labile fraction of cell wall phosphopeptidomannan	*

II. Glucan synthesis

GSC1 (W5Q_00233)	encoding beta-1,3-glucan synthase	*
IFF11 (W5Q_02548 and W5Q_02550	encoding a protein similar to HYR1p. Analysis of the *C. albicans* genome has identified the *IFF* gene family as encoding the largest family of cell wall-related proteins which is conserved in a wide range of *Candida* species. *IFF11* is important for maintaining the cell wall structure	(15), *
ALS2 (W5Q_05013), *ALS5* (W5Q_05030), *ALS6* (W5Q_03142) and *ALS7* (W5Q_03128 and W5Q_03143)	Agglutinin-like proteins are important cell wall proteins in *C. albicans* required for adhesion and biofilm formation	(16), *
PGA25 (W5Q_04597) and *PGA50* (W5Q_00609)	encode a probable GPI-anchored cell wall protein involved in cell wall organization, hyphal growth as well as virulence	*
HYR4 (W5Q_05538)	encoding a hyphally regulated cell wall protein 4	*
GCA1 (W5Q_00993) and *GCA12* (W5Q_01020)	genes encoding a glycosidase-like protein in the cell wall	*
CCW12 (W5Q_00875)	also known as *PGA6* Predicted GPI-anchored protein 6. The expression of probable cell wall proteins that participate in adhesive cell-cell interactions	*

III. *Ergosterol synthesis*

ERG3 (W5Q_00467)	encoding a sterol C5,6-desaturase which is essential for synthesis of ergosterol	*
ERG6 (W5Q_02705 and W5Q_02721)	encoding a methyltransferase	*
ERG252 (W5Q_03456)	encoding C-4 sterol methyl oxidase	*

10. *Streptococcus mutans* enhances *Candida albicans* cellular stress related gene responses

CTA1 (W5Q_00661)	encoding Catalase A was upregulated, protecting cells from the toxic effects of hydrogen peroxide stress	*
CCP1 (W5Q_02762)	encoding cytochrome-c peroxidase was upregulated which destroys radicals which are normally produced within the cells and which are toxic to biological systems	*
GPX2 (W5Q_00712)	encoding Gpx2p capable of reducing H_2O_2 and *tert*-butyl hydroperoxide in the presence of thioredoxin, thioredoxin reductase and NADPH were among the upregulated proteins	(17), *
POS5 (W5Q_03211)	encoding a mitochondrial NADH kinase involved in oxidative stress	*

11. *Streptococcus mutans* stimulated *Candida albicans* cellular reproduction

I. Mitotic cell division

STU1 (W5Q_00713)	encoding a microtubule binding protein that promotes the stabilization of dynamic microtubules and required for mitotic spindle formation	*
MAD1 (W5Q_01226)	encoding a spindle assembly checkpoint protein	*
CSE1 (W5Q_00745)	involved in chromosome segregation were among the overexpressed genes. *CSE1* and *CSE2* were identifies as two new genes required for accurate mitotic chromosome segregation in *S. cerevisiae*	(18), *
ASE1 (W5Q_03103)	anaphase spindle elongation protein	*
APC5 (W5Q_03823)	encoding an anaphase-promoting complex subunit 5	*
CDC27 (W5Q_05582)	encoding an anaphase-promoting complex subunit	*
SWI6 (W5Q_00835)	encoding a putative protein component of the transcription complexes termed MBF and SBF involved in G1/S cell-cycle progression	*
CAC2 (W5Q_04378)	encoding a chromatin assembly factor 1 subunit p60	*
CDC37 (W5Q_04837)	encoding cell cycle proteins necessary for passage in the cell division cycle	*
CDC4 (W5Q_05627), *BUB1* (W5Q_03598), *BUB31* (W5Q_04346)	Proteins important for cell division control	*
MEC1 (W5Q_04425)	proteins associated with cell cycle checkpoints	*

II. Meiotic cell division

MEK1 (W5Q_02173)	encoding a meiosis-specific serine/threonine-protein kinase	*
MEI5 (W5Q_06018)	encoding a protein showing weak similarity to *S. cerevisiae* protein required for synapsis and meiotic recombination	*
MND1 (W5Q_06176)	encoding a meiotic nuclear division protein 1	*
MSC1 (W5Q_06365)	encoding a meiotic sister chromatid recombination protein 1	*
RIM4 (W5Q_03616)	a positive regulator of sporulation-specific genes and of sporulation and is required for premeiotic DNA synthesis and meiotic chromosomal segregation	*

12. Several *Candida albicans* genes downregulated in the presence of *Streptococcus mutans*

I. Genes associated with cell membrane structure and function

POM33 (W5Q_00188)	encoding a pore membrane protein associated with cell membrane transport	*
TOM20 (W5Q_00254)	encoding an outer membrane translocase	*
FTR2 (W5Q_01374)	a plasma membrane iron permease gene	*
ERP3 (W5Q_01960) and *LSB5* (W5Q_02390)	encoding membrane trafficking proteins	*
SNL1 (W5Q_01017), *SUR7* (W5Q_04751)	genes encoding integral membrane proteins	*
YHU0 (W5Q_04051)	a *C. albicans* pH regulated GPI-anchored membrane protein	*
YL326 (W5Q_00305), *YG1I* (W5Q_06078), *YO073* (W5Q_03020) and *YD090* (W5Q_03275)	several uncharacterized membrane proteins	*

II. Mitochondrial membrane associated genes

YMC1 (W5Q_00699)	encoding a likely mitochondrial carrier protein	*
TOM37 (W5Q_01661)	encoding a translocase of the outer mitochondrial membrane	*
SHH3 (W5Q_01613) and *FMP42* (W5Q_05906)	encoding mitochondrial membrane proteins	*

III. Vacuolar membrane proteins

PFF1 (W5Q_00001)	a vacuolar membrane protease	*
VCX1 (W5Q_00826)	a calcium transport protein	*
PHM7 (W5Q_02235)	a potential transmembrane protein	*
YO292 (W5Q_06403)	encoding a vacuolar membrane protein	*
TVP18 (W5Q_00946), *TVP15* (W5Q_04011)	Membrane proteins in the golgi apparatus	*
SEC61 (W5Q_03289), *PBN1* (W5Q_04724) and *YET1* (W5Q_03342)	coding for an ER transmembrane protein	*

* Information from UniprotKB for *Candida albicans* database

1. Martin, R., Walther, A., and Wendland, J. (2005) Ras1-induced hyphal development in Candida albicans requires the formin Bni1. *Eukaryotic cell* 4, 1712-1724.

2. Zaragoza, O., Blazquez, M. A., and Gancedo, C. (1998) Disruption of the Candida albicans TPS1 gene encoding trehalose-6-phosphate synthase impairs formation of hyphae and decreases infectivity. *Journal of bacteriology* 180, 3809-3815.

3. Greig, J. A., Sudbery, I. M., Richardson, J. P., Naglik, J. R., Wang, Y., and Sudbery, P. E. (2015) Cell cycle-independent phospho-regulation of Fkh2 during hyphal growth regulates Candida albicans pathogenesis. *PLoS pathogens* 11, e1004630.

4. Kitanovic, A., Nguyen, M., Vogl, G., Hartmann, A., Gunther, J., Wurzner, R., Kunkel, W., Wolfl, S., and Eck, R. (2005) Phosphatidylinositol 3-kinase VPS34 of Candida albicans is involved in filamentous growth, secretion of aspartic proteases, and intracellular detoxification. *FEMS yeast research* 5, 431-439.

5. Woo, M., Lee, K., and Song, K. (2003) MYO2 is not essential for viability, but is required for polarized growth and dimorphic switches in Candida albicans. *FEMS microbiology letters* 218, 195-202.

6. Pérez-Martín, J., Uría, J. A., and Johnson, A. D. (1999) Phenotypic switching in Candida albicans is controlled by a SIR2 gene. *The EMBO Journal* 18, 2580-2592.

7. Zacchi, L. F., Gomez-Raja, J., and Davis, D. A. (2010) Mds3 regulates morphogenesis in Candida albicans through the TOR pathway. *Molecular and cellular biology* 30, 3695-3710.

8. Ding, X., Yu, Q., Xu, N., Wang, Y., Cheng, X., Qian, K., Zhao, Q., Zhang, B., Xing, L., and Li, M. (2013) Ecm7, a regulator of HACS, functions in calcium homeostasis maintenance, oxidative stress response and hyphal development in Candida albicans. *Fungal genetics and biology : FG & B* 57, 23-32.

9. Hayek, P., Dib, L., Yazbeck, P., Beyrouthy, B., and Khalaf, R. A. (2010) Characterization of Hwp2, a Candida albicans putative GPI-anchored cell wall protein necessary for invasive growth. *Microbiological research* 165, 250-258.

10. Nobile, C. J., Fox, E. P., Hartooni, N., Mitchell, K. F., Hnisz, D., Andes, D. R., Kuchler, K., and Johnson, A. D. (2014) A histone deacetylase complex mediates biofilm dispersal and drug resistance in Candida albicans. *mBio* 5, e01201-01214.

11. Ramanan, N., and Wang, Y. (2000) A high-affinity iron permease essential for Candida albicans virulence. *Science (New York, N.Y.)* 288, 1062-1064.

12. Chen, Y.-L., Kauffman, S., and Reynolds, T. B. (2008) Candida albicans Uses Multiple Mechanisms To Acquire the Essential Metabolite Inositol during Infection. *Infection and immunity* 76, 2793-2801.

13. Piekarska, K., Mol, E., van den Berg, M., Hardy, G., van den Burg, J., van Roermund, C., MacCallum, D., Odds, F., and Distel, B. (2006) Peroxisomal Fatty Acid β-Oxidation Is Not Essential for Virulence of Candida albicans. *Eukaryotic cell* 5, 1847-1856.

14. Ramirez-Zavala, B., Mottola, A., Haubenreisser, J., Schneider, S., Allert, S., Brunke, S., Ohlsen, K., Hube, B., and Morschhauser, J. (2017) The Snf1-activating kinase Sak1 is a key regulator of metabolic adaptation and in vivo fitness of Candida albicans. *Molecular microbiology* 104, 989-1007.

15. Bates, S., de la Rosa, J. M., MacCallum, D. M., Brown, A. J. P., Gow, N. A. R., and Odds, F. C. (2007) Candida albicans Iff11, a Secreted Protein Required for Cell Wall Structure and Virulence. *Infection and immunity* 75, 2922-2928.

16. Hoyer, L. L. (2001) The ALS gene family of Candida albicans. *Trends Microbiol* 9, 176-180.

17. Tanaka, T., Izawa, S., and Inoue, Y. (2005) GPX2, encoding a phospholipid hydroperoxide glutathione peroxidase homologue, codes for an atypical 2-Cys peroxiredoxin in Saccharomyces cerevisiae. *The Journal of biological chemistry* 280, 42078-42087.

18. Xiao, Z., McGrew, J. T., Schroeder, A. J., and Fitzgerald-Hayes, M. (1993) CSE1 and CSE2, two new genes required for accurate mitotic chromosome segregation in Saccharomyces cerevisiae. *Molecular and cellular biology* 13, 4691-4702.
